# A small molecule disrupts G4–STAT1 interaction and synergizes with olaparib to drive cancer cell death

**DOI:** 10.1093/nar/gkag383

**Published:** 2026-05-05

**Authors:** Yingying Wang, Xuenan Zhang, Yuting Bian, Sihan He, Rongshuang Cheng, Jinzhu Li, Jonathan Dickerhoff, Yushuang Liu, Yu Zhou, Jinlei Bian, Kewei Zheng, Danzhou Yang, Ling-Yi Kong, Kai-Bo Wang

**Affiliations:** State Key Laboratory of Natural Medicines, Joint International Research Laboratory of Target Discovery and New Drug Innovation (Ministry of Education), and Jiangsu Key Laboratory of Bioactive Natural Product Research, China Pharmaceutical University, Nanjing, Jiangsu 211198, PR China; School of Biomedical Sciences, Hunan University, Changsha, Hunan 410082, PR China; State Key Laboratory of Natural Medicines, Joint International Research Laboratory of Target Discovery and New Drug Innovation (Ministry of Education), and Jiangsu Key Laboratory of Bioactive Natural Product Research, China Pharmaceutical University, Nanjing, Jiangsu 211198, PR China; State Key Laboratory of Natural Medicines, Joint International Research Laboratory of Target Discovery and New Drug Innovation (Ministry of Education), and Jiangsu Key Laboratory of Bioactive Natural Product Research, China Pharmaceutical University, Nanjing, Jiangsu 211198, PR China; State Key Laboratory of Natural Medicines, Joint International Research Laboratory of Target Discovery and New Drug Innovation (Ministry of Education), and Jiangsu Key Laboratory of Bioactive Natural Product Research, China Pharmaceutical University, Nanjing, Jiangsu 211198, PR China; State Key Laboratory of Natural Medicines, Joint International Research Laboratory of Target Discovery and New Drug Innovation (Ministry of Education), and Jiangsu Key Laboratory of Bioactive Natural Product Research, China Pharmaceutical University, Nanjing, Jiangsu 211198, PR China; Department of Medicinal Chemistry and Molecular Pharmacology, Purdue University, West Lafayette, IN 47907, America; State Key Laboratory of Natural Medicines, Joint International Research Laboratory of Target Discovery and New Drug Innovation (Ministry of Education), and Jiangsu Key Laboratory of Bioactive Natural Product Research, China Pharmaceutical University, Nanjing, Jiangsu 211198, PR China; State Key Laboratory of Natural Medicines, Joint International Research Laboratory of Target Discovery and New Drug Innovation (Ministry of Education), and Jiangsu Key Laboratory of Bioactive Natural Product Research, China Pharmaceutical University, Nanjing, Jiangsu 211198, PR China; State Key Laboratory of Natural Medicines, Joint International Research Laboratory of Target Discovery and New Drug Innovation (Ministry of Education), and Jiangsu Key Laboratory of Bioactive Natural Product Research, China Pharmaceutical University, Nanjing, Jiangsu 211198, PR China; School of Biomedical Sciences, Hunan University, Changsha, Hunan 410082, PR China; Department of Medicinal Chemistry and Molecular Pharmacology, Purdue University, West Lafayette, IN 47907, America; State Key Laboratory of Natural Medicines, Joint International Research Laboratory of Target Discovery and New Drug Innovation (Ministry of Education), and Jiangsu Key Laboratory of Bioactive Natural Product Research, China Pharmaceutical University, Nanjing, Jiangsu 211198, PR China; State Key Laboratory of Natural Medicines, Joint International Research Laboratory of Target Discovery and New Drug Innovation (Ministry of Education), and Jiangsu Key Laboratory of Bioactive Natural Product Research, China Pharmaceutical University, Nanjing, Jiangsu 211198, PR China

## Abstract

Bloom syndrome protein (BLM), a RecQ family DNA helicase, is consistently overexpressed in multiple malignancies, yet its therapeutic potential remains largely unexplored. Herein, we focused on targeting the *BLM* promoter G-quadruplex (*BLM*-G4) to inhibit the *BLM* signaling pathway. We first characterized the parallel *BLM*-G4 in the *BLM* promoter region. Subsequently, it is shown for the first time that *BLM*-G4 recruits phosphorylated signal transducer and activator of transcription 1 (pSTAT1) to activate *BLM* expression. Importantly, two natural alkaloids, berberine (BER) and coptisine (COP), compete with STAT1 for binding to *BLM*-G4, thereby significantly suppressing *BLM* expression in colon cancer cells. The BER/COP−*BLM*-G4 complex structures were determined using nuclear magnetic resonance experiments, which provide valuable insights for the rational design of next-generation *BLM*-G4-targeting ligands. Beyond *BLM* regulation, the conjoint analysis of genome-wide STAT1-CUT&Tag-seq, G4-CUT&Tag-seq, and COP-RNA-seq demonstrated STAT1 as a general G4-binding transcription factor and COP as a pan-genomic G4 stabilizer. Furthermore, BER/COP exhibited a pronounced synergistic effect with olaparib in inducing colon cancer cell death by disrupting DNA repair pathways and intensifying DNA damage. Collectively, our findings reveal a novel epigenetic mechanism of *BLM* gene upregulation mediated by *BLM*-G4−STAT1 interaction and suggest that the combination therapy of G4 stabilizers with poly(ADP) ribose polymerase (PARP) inhibitors is a promising strategy for treating complex cancers.

## Graphical Abstract

**Graphical Abstract:**
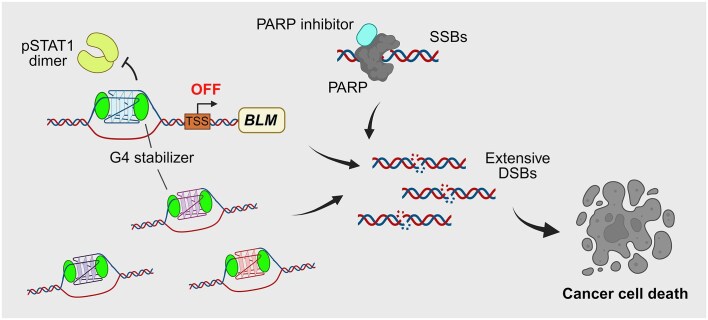

## Introduction

BLM (Bloom syndrome protein), a member of the RecQ family DNA helicases, plays a crucial role in unwinding various aberrant nucleic acid secondary structures, such as G-quadruplexes (G4s), DNA displacement loops (D-loops), and Holliday junctions, and thus suppresses aberrant recombination at these sites and maintains genome stability [1, 2]. Additionally, BLM is characterized as a critical upstream component for the recruitment of homologous recombination (HR) and canonical non-homologous end joining (c-NHEJ) factors to DNA double-strand break (DSB) sites, balancing the cellular DNA repair process by exerting both pro- and anti-recombinogenic functions [3]. Moreover, BLM is reported to negatively regulate the error-prone microhomology-mediated end joining (MMEJ), a particular form of alternative NHEJ (alt-NHEJ) pathway, to prevent the erroneous repair of DSBs [4]. Loss-of-function mutations in BLM can lead to Bloom syndrome (BS), characterized by growth retardation, immune deficiency, and cancer susceptibility. However, recent studies have revealed a strong association between BLM overexpression and cancer progression [1].

Compared with normal tissues, BLM is overexpressed in various types of cancer tissues [5, 6]. *In vitro* studies have demonstrated that the overexpressed BLM can displace single-stranded DNA (ssDNA)-binding proteins (RAD51 or RPA) and compress ssDNA into phase-separated condensates, which possibly contributes to cancer progression [7]. Additionally, BLM helicase unwinds DNA lagging strand substrates to facilitate the assembly of the ALT (alternative lengthening of telomeres) damage response, thus ensuring the malignant proliferation of cancer cells originating from mesenchymal tissues [8]. Clinically, BLM overexpression is closely linked to drug resistance and poor survival of patients, making BLM a potential prognostic marker of advanced cancers [5, 6, 9, 10]. BLM has also been identified as one of the top 50 targets associated with DNA damage response (DDR) in cancer therapy [11]. Correspondingly, small molecules targeting the BLM protein were designed and synthesized to inhibit cancer progression [12–14]. However, none of the BLM-targeting drugs has yet entered clinical trials. Therefore, we focused on targeting *BLM* proximal promoter G-quadruplexes (G4s) as an alternative strategy to destroy BLM-implicated DNA repair pathways for cancer therapy.

G4s, non-canonical nucleic acid secondary structures, are well known for their regulatory roles in DNA replication, transcription, translation, and epigenetics [15–17]. They are enriched in cancer-related genes and predominantly form in cancer cells rather than in normal cells [18–21]. The G4s in oncogene promoters can recruit various regulatory proteins, including helicases, polymerases, transcription factors, and topological proteins, to regulate oncogene expression levels, thus altering cellular biological processes and contributing greatly to cancer development [22–24]. However, small molecules can target these abnormally formed G4s and block DNA replication and transcription, thereby effectively killing cancer cells [25–27]. As a representative example, the successful entrance of CX-5461 (a G4-binding ligand) into the Phase I clinical trial has made G4s highly pursued drug targets [28]. Nevertheless, the recently reported mutagenic effect of CX-5461 [29, 30] emphasizes that continuous efforts are needed to develop safe and effective clinical G4-targeting drugs.

Previous works have identified the potential G4-forming sequence in the *BLM* proximal promoter region and demonstrated its formation as parallel G4s with transcription-promoting functions [31]. Recently, we determined the structure of a cGMP-fill-in oxidation-induced vacancy G4 in the *BLM* promoter region and found that targeting *BLM* promoter G4s was a promising strategy for *BLM* signaling inhibition [32]. However, the detailed structure, functions, and therapeutic applications of the native *BLM* promoter G4 (*BLM*-G4) have yet to be fully elucidated.

In this study, we first characterized the native *BLM*-G4 in the *BLM* proximal promoter region and demonstrated that *BLM*-G4 recruited Signal Transducer and Activator of Transcription 1 (STAT1) to upregulate *BLM* gene expression. Subsequently, we found that the canonical G4-binding alkaloids, berberine (BER) and coptisine (COP), could downregulate *BLM* gene expression by binding to *BLM*-G4 in competition with STAT1. To gain the structural insights into *BLM*-G4 and its complexes with BER/COP, their solution complex structures were deciphered through nuclear magnetic resonance (NMR) spectroscopy and AMBER calculations, which provide an impelling structural basis for the design of *BLM*-G4-targeting ligands. Beyond *BLM* regulation, the conjoint analysis of genome-wide STAT1-CUT&Tag-seq, G4-CUT&Tag-seq, and COP-RNA-seq demonstrated STAT1 as a general G4-binding transcription factor and COP as a pan-genomic G4 stabilizer which downregulated numerous G4-containing genes associated with genetic information processing. Moreover, there was a significant synergistic effect between BER/COP and olaparib (OLP) in inducing DSBs, which exacerbated the death of colon cancer cells. In summary, our study reveals a novel epigenetic mechanism of *BLM* gene upregulation mediated by *BLM*-G4–STAT1 interaction and demonstrates that the drug combination of G4 stabilizers with poly(ADP) ribose polymerase (PARP) inhibitors is a promising therapeutic strategy against complex cancers.

## Materials and methods

### Sample preparation

DNA oligonucleotides were purchased from Sangon Biotech Co., Ltd (China). The sequences are listed in Supplementary Table S1. DNA oligonucleotides were dissolved in buffer (50 mM K_2_HPO_4_/KH_2_PO_4_, pH 7.0, 10/90% D_2_O/H_2_O), heated at 95°C for 5 min, and cooled down slowly to room temperature for G4 formation. COP and BER were purchased from Standard Technology (ST071901, ST030701, China). OLP was purchased from APExBIO (A4154, America). The compounds were dissolved in dimethylsulfoxide (DMSO) at 40 mM.

### Circular dichroism experiments

The determination of circular dichroism (CD) spectra and melting curves was conducted according to the reported protocol [33] with some modifications. CD data were collected by a Jasco-1500 spectropolarimeter (Japan). DNA samples (20 μM) were prepared in the K_2_HPO_4_/KH_2_PO_4_ buffer containing 0.1/10/50 mM K^+^ at pH 7.0. The DNA samples were annealed before testing. BER or COP was incubated with the annealed G4 DNA (0.1 mM K^+^ condition) in gradient concentrations for 3 h. CD spectra were collected at 25°C by using a 1 mm path length quartz cuvette. The pure buffer was measured for blank correction. For CD melting experiments, the sample was heated from 25 to 95°C with a heating rate of 2°C/min, and the CD ellipticity at 264 nm was constantly recorded. The melting temperature (*T*_m_) was determined as the intersection temperature at the median value of the melting curve.

### Native electrophoretic mobility shift assay 

The electrophoretic mobility shift (EMSA) experiment was conducted according to the previous protocol [33] with some modifications. DNA samples (150 μM), with or without 4 equivalents of BER and COP, were annealed in a buffer containing 50 mM K_2_HPO_4_/KH_2_PO_4_, pH 7.0. A 10 × 7 cm native gel (containing 12.5 mM KCl and 16% acrylamide, pH 8.0) was used. The gel electrophoresis was conducted at 45 V for 2 h in 1× TBE buffer containing 12.5 mM KCl. DNA bands were visualized under 254 nm UV light.

### Nuclear magnetic resonance spectroscopy

The NMR spectra were collected on a Bruker AV-600 spectrometer equipped with a QCI cryoprobe (America). The w5 water suppression was used. 2D NOESY spectra were collected at temperatures of 15, 25, and 35°C with mixing times of 80 and 400 ms, respectively. DQF-COSY and ^1^H-^13^C HSQC spectra were collected at 25°C. Data were analyzed and processed using Topspin 3.5 (Bruker) and Sparky (UCSF) software. Chemical shift calibration was done indirectly for ^13^C relative to the internal standard 4,4-dimethyl-4-silapentane-1-sulfonic acid (DSS) and directly for ^1^H according to the water signal relative to DSS.

### Structural calculation

The 10 lowest-energy solution structures of *BLM*-G4, BER–*BLM*-G4, and COP–*BLM*-G4 were calculated by Xplor-NIH and AMBER incorporating 2D NOESY information, in reference to the previously reported methods for structure elucidation and calculation [33].

### Fluorescence titration assay

Fluorescence data were collected on a Jasco-FP8300 spectrofluorometer (Japan). The emission spectra were acquired from 520 to 600 nm in a 1 cm path length quartz cell. The excitation wavelength was set at 377 nm. BER or COP concentrations were fixed at 0.2 μM in 50 mM K^+^ solution. Gradient concentrations of *BLM*-G4 were added sequentially. Fluorescence spectra were collected after 2 min incubation at each titration. *K*_d_ values were calculated through an equation based on 2:1 (drug:G4) binding stoichiometry:


(1) \begin{eqnarray*}F &=& {{F}_{min}} \,{+}\, \left( {{{F}_{max}} \,{-}\, {{F}_{min}}} \right)\!\left[\! {\left( {2{{D}_T} \,{+}\, {{C}_T} \,{+}\, {{K}_d}} \right) \,{-}\, {{{\left(\! {{{{\left( {2{{D}_T} \,{+}\, {{C}_T} \,{+}\, {{K}_d}} \right)}}^2} \,{-}\, \left( {8{{D}_T}{{C}_T}} \right)\!} \right)}}^{1/2}}} \right]/( {2{{C}_T}} ) \\ \end{eqnarray*}

where *F* is the ligand-induced fluorescence intensity at 530 nm; ${{C}_T}$ is the ligand concentration; and${{D}_T}$ is the ternary complex concentration.

### DNA polymerase stop assay

The DNA polymerase stop assay (DPSA) was carried out as described, with some modifications [21]. The 5′-end FAM-labeled primer was mixed with *BLM*-G4 template DNA (5′-T**CACAAAGAGGAGGGAGGGCGGGAGGGAAGGAGAGA**TAGCTGCACGCAATTGCTATAGTGAGTCGTATTA-3′, bold letters represent the endogenous promoter sequence) or non-G4 template DNA in a 1.2:1 ratio (Supplementary Table S1). The mixtures with various concentrations of BER, COP, or K^+^ were annealed by heating at 95°C for 5 min, then slowly cooled to room temperature. Primer extension was conducted for 10 min in 50 μl reaction buffer containing 0.12 μM DNA template, 0.144 μM FAM-labeled primer, 100 μM dNTP, 0.5 U of Klenow fragment (exo-) DNA polymerase (EP0422, Thermo Scientific, America), 1 mM KCl, 1 mM dithiothreitol (DTT), and 10 mM MgCl_2_. The DNA products were loaded onto a 12% denatured polyacrylamide gel, then electrophoresis was conducted at 100 V for 60 min at room temperature. DNA fragments were visualized on a GelDoc Go Imager (BIO-RAD, America) and processed by Image Lab 4.0.

### Cell culture

H1975 and HCC-LM3 cells were cultivated in RPMI 1640 medium (containing 10% bovine serum and 1% penicillin–streptomycin). H1299, H226, H460, MKN-74, and NCI-N87 cells were cultivated in RPMI 1640 medium (containing 10% bovine serum, 1% penicillin–streptomycin, and 1% sodium pyruvate). HCT116, HT-29, MCF-10A, MCF-7, MDA-MB-231, HepG2, Huh-7, BEAS-2B, and A549 cells were cultivated in Dulbecco’s modified Eagle’s medium (DMEM; containing 10% bovine serum and 1% penicillin-streptomycin). Hep3B cells were cultivated in minimum essential medium (MEM; containing 10% bovine serum and 1% penicillin–streptomycin).

### Dual-luciferase reporter assay and quantitative PCR

**Figure 1.**
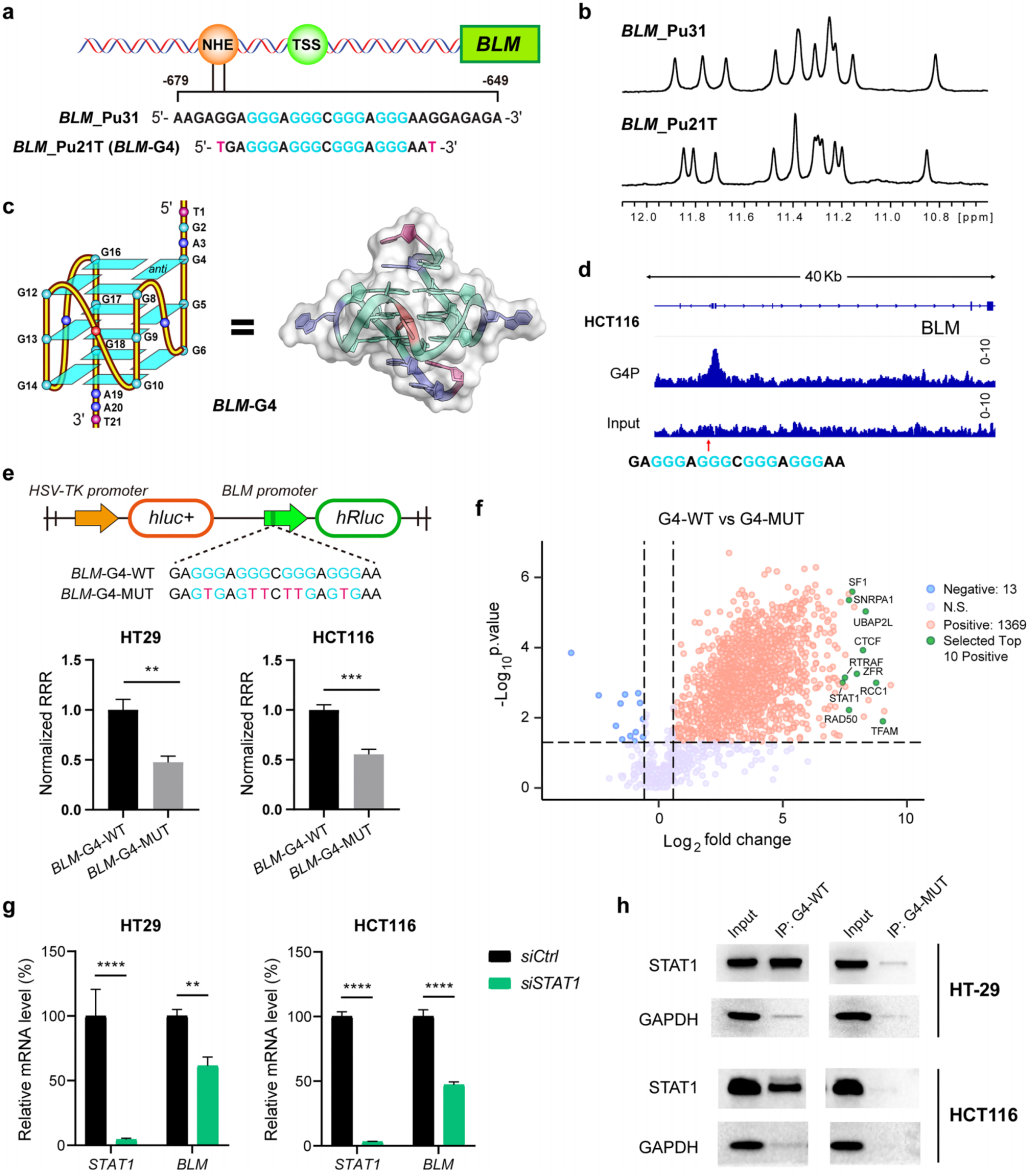
Native *BLM* promoter G4 recruits STAT1 to upregulate *BLM* gene expression. (**a**) The schematic of the human *BLM* gene promoter. The WT G4-forming sequence *BLM*_Pu31 and its truncated derivative *BLM*_Pu21T (*BLM*-G4) are shown. NHE, nuclease-hypersensitive element; TSS, transcription start site. (**b**) The ^1^H-NMR imino proton spectra of *BLM*_Pu31 and *BLM*_Pu21T. Conditions: 150 μM DNA, pH 7.0, 50 mM K^+^ solution, 25°C. (**c**) The schematic model of *BLM*-G4, as well as its solution structure in cartoon mode. (**d**) The enrichment of *BLM* promoter G4 by G4P in HCT116 cells. G4P ChIP-seq data were downloaded from GSE239694. (**e**) The upregulation of Renilla luciferase activity in the presence of the wild-type *BLM* promoter (*BLM*-G4-WT) in comparison with *BLM*-G4-MUT, as detected by dual-luciferase reporter assay. RRR (relative response ratio) = (Renilla response)/(Firefly response). *hluc+, Firefly luciferase* gene; *hRluc, Renilla luciferase* gene. (**f**) The volcano plot depicts the quantified fold changes (FCs) of various proteins pulled down by *BLM*-G4-WT versus *BLM*-G4-MUT sequences in HT-29 cell lysates. The selected top 10 regulatory protein candidates with the highest FCs are marked with green dots. N.S., not significant. (**g**) Relative transcription levels of *BLM* after knocking down STAT1 for 48 h in both HT-29 and HCT116 cells. (**h**) STAT1 protein in HT-29 and HCT116 cells was specifically pulled down by the *BLM*-G4-WT, rather than by the *BLM*-G4-MUT sequence. Statistical data are shown as the means ± SD of three independent experiments. *P*-values (**P < 0.01; ***P < 0.001; ****P < 0.0001) were determined by one-way ANOVA with post-hoc Dunnett’s test.

The *BLM* promoter sequence [−1000 to +50 bp relative to the transcription start site (TSS)] was inserted, replacing the SV40 promoter (KpnI–NheI restriction sites) of the psiCHECK2 vector to obtain psiCHECK2-*BLM*-G4-WT and psiCHECK2-*BLM*-G4-MUT plasmids. The wild-type (WT) or mutant (MUT) *BLM* promoter sequences, which varied at −674 to −656 bp relative to the TSS, are shown in Fig. 1e. The two recombinant plasmids were respectively transformed into HCT116 or HT-29 cells using Lipofectamine™ 3000 (L3000015, Thermo Scientific, America) according to the protocol. After 9 h of transfection, the cells were supplemented with fresh DMEM or 40 μM COP and cultured for 36 h. Then, the cells were collected and detected for their dual-luciferase activities according to the protocol of the Dual Luciferase Reporter Assay Kit (DL101, Vazyme, China). Alternatively, after 36 h of cultivation, cell RNA was extracted with DNase I used to digest genomic DNA and plasmid DNA, which was then reverse-transcribed into cDNA for quantitative PCR (qPCR) analysis. Primers used for dual luciferase qPCR are listed in Supplementary Table S3.

### *BLM*-G4 pulldown

Biotin-labeled *BLM*-G4-WT and *BLM*-G4-MUT oligonucleotides dissolved in buffer (50 mM K_2_HPO_4_/KH_2_PO_4_, pH 7.0) were denatured at 90°C for 5 min and annealed naturally overnight. Three 10 cm dishes of HT-29 cells at 80% confluency were lysed with RIPA lysis buffer for 40 min on ice. The lysed cells were collected and centrifuged to obtain the protein supernatants, which were then quantified by the BCA quantification kit (WB6501, NCM Biotech, China). The total protein supernatant was divided into two aliquots for each dish, with each aliquot containing 1 mg of proteins. They were firstly pre-cleared by incubating with 200 μl of streptavidin agarose 6FF (HY-K0218A, MCE, America) at 4°C for 1 h. After centrifugation at 1000 rpm for 5 min, the two aliquots of protein supernatants were each incubated with 15 nmol *BLM*-G4-WT or *BLM*-G4-MUT (three biological repeats) by rotating at 4°C overnight. Then, they were each incubated with another 200 μl of pre-washed streptavidin agarose 6FF by rotating at 4°C for 4 h. The beads were washed with phosphate-buffered saline (PBS) for five times, then subjected to liquid chromatography–tandem mass spectrometry (LC-MS/MS) analysis. The top 10 candidate regulatory proteins with the highest pulldown fold changes (FCs; *BLM*-G4-WT versus *BLM*-G4-MUT) were chosen for further analysis.

For the G4 pulldown–western blot experiment, 20 μM biotin-labeled *BLM*-G4-WT and *BLM*-G4-MUT oligonucleotides were annealed as mentioned above, then incubated with five equivalents of COP/BER for 10 h at room temperature. DMSO was used as the control. The drug–G4 complexes were incubated with pre-cleared proteins (extracted from HT-29 or HCT116 cells) at 4°C overnight, followed by incubating with pre-washed streptavidin agarose 6FF at 4°C for 4 h. After washing five times with PBS, the agarose bead complexes were resuspended in 40 μl of 2× Laemmli buffer and boiled at 95°C for 10 min. The supernatants were then subjected to western blot analysis.

### Small interfering RNA knockdown

Cells were seeded into 6-well plates at 5 × 10^5^ cells per well and incubated for 24 h. Small interfering RNA (siRNA) was incubated with CALNP™ RNAi *in vitro* (DN001, D-Nano Therapeutics, China) in fresh medium according to the protocol. Then, the siRNA mixtures were added into cells for transfection. Total RNA was extracted after 48 h of transfection. Total proteins were extracted after 60 h of transfection. siRNA sequences are listed in Supplementary Table S2. qPCR was conducted as described below, and the primers are listed in Supplementary Table S3.

### Heterologous expression and purification of STAT1

Following previous studies [34, 35], the *Escherichi coli* TKB1 strain which expressed the tyrosine kinase Elk was chosen for the heterologous expression of phosphorylated STAT1 (pSTAT1). Firstly, the pET28a plasmid encoding the STAT1 core domain (STAT1c, residues S132–H713) with an N-terminal 6× His tag was transformed into the *E. coli* TKB1 strain. Protein expression was induced with 0.2 mM isopropyl-β-d-thiogalactopyranoside (IPTG) for 16 h at 16°C. After induction, cells were harvested and resuspended in tyrosine kinase induction medium [M9 medium supplemented with 1 mM MgSO_4_, 11 mM glucose, 0.1% (w/v) casamino acids, 1.5 µM thiamine-HCl, and 53 µM indole-acrylic acid] and grown for an additional 2.5 h at 37°C to facilitate phosphorylation of STAT1c (pSTAT1c). Pelleted cells were lysed using Super bacterial lysis buffer (BR0005-02, ACE, China), followed by purification using BeyoGold™ His-tag Purification Resin (P2218, Beyotime, China). Eluted fractions were alkylated by adding 20 mM *N*-ethylmaleimide to avoid protein aggregation due to cysteine cross-linking at high concentrations of proteins. The alkylation reaction was terminated by adding 50 mM β-mercaptoethanol. Protein samples were washed and concentrated using Amicon Ultra-15 concentrators (UFC905008, Millipore, America). STAT1c monomer was purified using the same plasmid similarly induced in the *E. coli* BL21(DE3) strain, followed by the same purification strategy without protein phosphorylation. Size exclusion chromatography was conducted at 4°C using Superdex™ 200 Increase 10/300 GL (Cytiva, America).

### SDS–PAGE

For sodium dodecylsulfate–polyacrylamide gel electrophoresis (SDS–PAGE) analysis, the protein samples were mixed with 5× loading dye, then loaded onto the 8% denatured polyacrylamide gel. Electrophoresis (140 V for 1 h) was conducted at room temperature. The gel was stained with Coomassie Brilliant Blue G250 (P0003M, Beyotime, China) for 30 min and the background was washed with pure water.

### STAT1–G4 EMSA

FAM-labeled *BLM*-G4, *BLM*_Pu31, *BLM*-G4-mut, and *GAS* duplex (0.1 μM) were respectively incubated with different concentrations of pSTAT1c/STAT1c in 10 μl of binding buffer [20 mM Tris–HCl, pH 7.5, 2 mM MgCl_2_, 2 mM DTT, 100 mM KCl, 1 mM EDTA, 0.4 mg ml^−1^ bovine serum albumin (BSA)] for 1 h at room temperature. Then, 10 μl of each sample was loaded onto the 6% native polyacrylamide gels, followed by electrophoresis (100 V for 1 h) in 1× TBE buffer containing 100 mM K^+^ in an ice bath. The gels were visualized on a GelDoc Go Imager (BIO-RAD, America), and the images were processed by Image Lab 4.0. For competitive EMSA, pSTAT1c (5 μM) was co-incubated with FAM-labeled *BLM*_Pu31 (0.1 μM) and different competitive DNAs (5 μM) for 1 h at room temperature, then taken electrophoresis. For drug–G4–STAT1 EMSA, *BLM*-G4 (0.1 μM) was pre-incubated with different concentrations of BER/COP for 3 h at room temperature, then with pSTAT1c (5 μM) for 1 h, followed by electrophoresis.

### Cell viability assay

Cells were seeded into 96-well plates at 2 × 10^3^ cells per well and cultured for 24 h, then treated with different concentrations of drugs for 48 and 72 h. Cell viability was measured by CCK-8 detection reagent (K1018, APExBIO, America) at 450 nm wavelength.

### Quantitative PCR

Cells under 48 h treatment with different concentrations of drugs were collected for RNA extraction (RNA-Quick Purification Kit, RN001, Yishan Biotech, China). RNA was then reverse transcribed into cDNA (ABScript III RT Master Mix for qPCR with gDNA Remover, RK20429, ABclonal, China). 2× Universal SYBR Green Fast qPCR Mix (RK21203, ABclonal, China) was used for qPCR which was conducted on a LightCycler 480 (Roche, Germany). The qPCR primers are listed in Supplementary Table S3. The data were analyzed through the 2^−ΔΔCt^ method and plotted by GraphPad Prism 8.

### Western blotting

Cells treated with different concentrations of drugs for 48 h were collected and lysed on ice for 40 min using RIPA lysis buffer. The cell lysates were centrifuged (12 000 rpm, 10 min, 4°C) to obtain the protein supernatant, which was subsequently quantified by a BCA quantification kit, followed by boiling for 10 min for denaturation. Gel electrophoresis was conducted using an 8% (or 15%) denatured polyacrylamide gel (80 V for 2 h). The target gel bands were electrotransferred onto a 0.2 μm polyvinyldifluoridene (PVDF) membrane (300 mA for 1 h). Then, the membranes were blocked with 5% skim milk for 2 h, washed with Tris-buffered saline–Tween (TBST) three times for 10 min, and incubated with the primary antibodies against glyceraldehyde phosphate dehydrogenase (GAPDH; A19056-Rabbit, ABclonal, China), BLM (SC-365753-Mouse, Santacruz, Germany), STAT1 (14995S-Rabbit, Cell Signaling Technology, America), pSTAT1 (Tyr701) (9167S-Rabbit, Cell Signaling Technology, America), or γ-H2AX (AP0687-Rabbit, ABclonal, China) at 4°C overnight. After washing with TBST (3 × 10 min), the membranes were incubated with horseradish peroxidase (HRP)-conjugated goat anti-mouse (or rabbit) secondary antibody (AS003/AS014, ABclonal, China) for 2 h at room temperature, followed by further TBST washes (3 × 10 min). Enhanced chemiluminescence (ECL) substrates were used for protein exposure on the ChemiDOC XRS + system (BIO-RAD, America). The results were analyzed by Image Lab 4.0.

### STAT1-CUT&Tag-seq

The CUT&Tag kit for Illumina was purchased from Ruoyu Biotech (CUT-01, China). Detection of STAT1-binding sites on chromosomal DNA in HCT116 cells was performed according to the kit protocols. Briefly, a total of 2 × 10^5^ cells were washed twice with 100 μl of 1× suspension buffer supplemented with protease inhibitors, and then resuspended with 100 μl of 1× suspension buffer. Next, 5 μl of pre-activated concanavalin A-coated magnetic beads were added to the cells and incubated on a rotator for 10 min at room temperature. The supernatant was removed, and the bead-bound cells were resuspended in 100 μl of 1× Dig-binding buffer (1× wash buffer containing 0.05% digitonin, 0.1% BSA, 2 mM EDTA, and 1 × protease inhibitors) containing 5 μg ml^−1^ recombinant anti-STAT1 antibody (ab234400-Rabbit, Abcam, Britain). The sample supplemented with non-specific rabbit IgG (2729S, Cell Signaling Technology, America) was used as the negative control. After 1 h rotation at room temperature, cells were washed with 100 μl of 1× Dig-binding buffer and resuspended with 100 μl of 1× Dig-binding buffer containing 1 μl of goat anti-rabbit IgG (Ruoyu Biotech, China), then incubated for another 1 h at room temperature. After washing three times with 1× Dig-wash buffer (1× wash buffer containing 0.05% digitonin and 1× protease inhibitors), a 1:110 dilution of pAG-Tn*5* + ME (mosaic end) adaptor was prepared in 100 μl of 1× Dig-binding buffer and added to the cells, which were then rotated for 1 h at room temperature. After four washes with 1× Dig-wash buffer, the bead-bound cells were resuspended in 50 μl of Fragmentation buffer (1× Dig-wash buffer containing 10 mM MgCl_2_) and incubated at 37°C for 1 h. To terminate the fragmentation reaction, 2.5 μl of 0.5 M EDTA and 1 μl of 20 mg ml^−1^ proteinase K were added and incubated at 55°C for 30 min, then incubated at 70°C for 20 min to inactivate the proteinase. The DNA was then extracted using 120 μl of DNA purification beads (Ruoyu Biotech, China) for library preparation. To amplify the libraries, 16 μl of purified DNA was mixed with a pair of uniquely barcoded i5 and i7 primers and amplified using 2× HG Library Mix (Ruoyu Biotech, China). Amplification was conducted with different cycles for STAT1 (18 cycles) and IgG (22 cycles) groups. The libraries were purified with 1.2× volumes of DNA purification beads and eluted with 20 μl of TE buffer for Illumina sequencing. Experiments were performed in duplicates.

The clean paired-end sequencing data were analyzed as described [36]. The human genome (version hg38) was used as the reference. Reads were aligned with bowtie2 (v.2.3.5.1) using –fast-local [37]. The coverage bigWig files were generated by deepTools (v3.3.2) bamCoverage using parameters –binSize 50 –extendReads –ignoreDuplicates [38].

### RNA sequencing

HCT116 cells were seeded into 6 cm dishes at 1 × 10^6^ cells per dish and cultivated for 24 h, then treated with 60 μM COP for 24 h. Subsequently, cell total RNA was extracted by the TRIzol method. Oligo(dT) was used to enrich mRNA, which was then fragmented and reverse-transcribed into cDNA. The adaptors were ligated to the ends of cDNA, then purified, sorted, and amplified to obtain the cDNA library. The library was sequenced on an Illumina platform (Sanshu Biotechnology, China).

### Cell-viability-based drug combination assay

Cells were seeded into 96-well plates at 2 × 10^3^ cells per well and cultivated for 24 h, then treated with different combinatorial concentrations of drugs for 48 or 72 h. Cell viability was measured by CCK-8 detection reagent at a wavelength of 450 nm. The ZIP (zero interaction potency) synergy score was calculated at the SynergyFinder+ online tool to evaluate the synergistic effects of combinatorial drugs, based on the ZIP model. The planar synergy plots were also generated at SynergyFinder+ .

### Alkaline comet assay

HT-29 cells treated with different concentrations of drugs for 48 h were digested and collected using 0.25% trypsin buffer, then washed and resuspended with PBS. Cells were added into 0.7% low melting point agarose, mixed, and tiled onto a glass microscope slide pre-coated with a layer of 1% normal melting point agarose gel. Slides were immersed in RIPA lysis buffer at 4°C for 2 h, then washed with PBS for 3 min. Alkaline electrophoresis buffer [containing 200 mM NaOH and 1 mM EDTA (pH 8.0), pH ~13] was pre-cooled at 4°C, then slides were immersed in the electrophoresis buffer at room temperature for 20 min. Horizontal electrophoresis was conducted at 25 V for 20 min in an ice bath. Then, slides were washed in neutralization buffer (400 mM Tris–HCl, pH 7.5) twice for 5 min each, followed by propidium iodide staining for 15 min. After washing three times with ultrapure water, the slides were photographed on the ImageXpress Micro Confocal microscope (America) under 10× objective. Tail moments were quantified using the CaspLab software. At least 50 cells per sample were analyzed. Quantitative data are presented as the mean ± standard deviation (SD) of three biological replicates.

### Immunofluorescence assay

HT-29 cells were seeded into the 96-well plates (6055300, Revvity, America) at 1× 10^4^ cells per well and incubated at 37°C for 24 h. After 48 h of drug treatment, cells were pre-washed with PBS, then fixed in 4% (w/v) paraformaldehyde for 10 min. After 3 × 3 min washes with PBS, cells were permeabilized in 0.5% Triton for 15 min, and washed with PBS for 3 × 3 min. Next, cells were blocked by 5% BSA for 1 h, washed with PBS for 3 × 3 min, then incubated with the mixtures of FAM-labeled G4P (1:200, synthesized from Synpeptide Biotech, China) and anti-γ-H2AX antibody (1:400, AP0687-Rabbit, ABclonal, China) at 4°C overnight. Subsequently, cells were washed with PBS for 3 × 10 min, then incubated with Alexa Fluor™ 594-conjugated donkey anti-rabbit secondary antibody (A-21207, Invitrogen, America) for 1 h at room temperature, and washed with PBS for 3 × 10 min. Following this, cells were counterstained with 4′,6-diamidino-2-phenylindole (DAPI) for 10 min at room temperature, washed with PBS for 3 × 3 min, and then 100 μl of PBS was added for photographing. G4P and γ-H2AX fluorescence was detected with a 40× objective lens on the Opera Phenix High Content Imaging System (PerkinElmer, America) through 488 and 594 nm passages, respectively. The mean fluorescence intensities of G4P and γ-H2AX in cell nuclei were analyzed using Harmony software. Quantitative data are presented as the mean ± SD from at least three independent experiments.

### Statistical analysis

GraphPad Prism 8 was used to plot all charts. The statistical significance was calculated using Student’s *t*-test or analysis of variance (ANOVA; one or two way), with post-hoc tests dependent on application (ns, not significant; *P < 0.05; **P < 0.01; ***P < 0.001; ****P < 0.0001).

## Results

### *BLM* promoter G4 recruits STAT1 to upregulate *BLM* gene expression

The native *BLM*-G4-forming sequence is located at the position from −679 to −649 bp relative to the TSS of the *BLM* promoter [31]. To characterize the biophysical properties of native *BLM*-G4, we conducted a series of *in vitro* experiments on the G4-forming sequences of wild-type *BLM*_Pu31 and its truncated derivative *BLM*_Pu21T (*BLM*-G4) which was designed to reduce the effect of the long flanking sequences (Fig. 1a). The presence of 12 similar benign imino proton peaks in ^1^H-NMR spectra indicated that *BLM*_Pu31 and *BLM*_Pu21T adopted the same three-tetrad G4 core (Fig. 1b). CD experiments further demonstrated their identical structural properties of forming parallel G4 topologies (Supplementary Fig. S1a), with the structural model shown in Fig. 1c. Additionally, CD melting curves revealed their high thermal stability (*T*_m_ > 50°C under 10 mM K^+^ buffer; Supplementary Fig. S1b). Since *BLM*_Pu21T (*BLM*-G4) showed higher thermal stability than *BLM*_Pu31 (Supplementary Fig. S1b), with shorter flanking sequences to reduce wobbling, it was used as the model sequence for further biophysical analysis (Fig. 1c).

Since BLM overexpression contributed greatly to tumor malignancy and drug resistance [1, 9, 10], we decided to investigate the potential role of *BLM*-G4 in regulating *BLM* gene expression. Firstly, the expression levels of BLM in various tumor cells were determined to identify the cancer types associated with high BLM expression. The results showed that BLM was highly expressed in HCT116, HT-29, MCF-7, MDA-MB-231, H1299, and MKN-74 tumor cells (Supplementary Fig. S2a). Considering that colon cancer has become the second leading cause of cancer-related deaths worldwide since 2023 (according to the World Health Organization), we selected HT-29 and HCT116 cancer cell lines for further study. The reported G4P ChIP-seq data (GSE239694) confirmed the existence of *BLM*-G4 in HCT116 cells (Fig. 1d) [39]. To characterize the potential transcription-regulatory role of *BLM*-G4 in the promoter, a dual-luciferase reporter assay was conducted in HT-29 and HCT116 cells. The SV40 promoter in the psiCHECK-2 plasmid was replaced with the *BLM* promoter sequence containing *BLM*-G4 or its mutant (*BLM*-G4-WT versus *BLM*-G4-MUT) (Fig. 1e) to launch the expression of the downstream Renilla luciferase. As a result, in comparison with the* BLM*-G4-MUT group, *BLM*-G4-WT significantly promoted the transcription and activity of the Renilla luciferase, which implied *BLM*-G4’s upregulatory role in *BLM* transcription (Fig. 1e; Supplementary Fig. S2b).

G4s in gene promoters normally recruit regulatory proteins to modulate downstream gene expression levels [23]. To identify the critical regulatory protein involved in *BLM*-G4-mediated transcriptional upregulation, a *BLM*-G4 pulldown experiment was conducted on HT-29 colon cancer cells. 3′-TEG-biotin-conjugated *BLM*-G4-WT and *BLM*-G4-MUT DNA sequences (Supplementary Table S1) were each incubated with total proteins from HT-29 cell supernatant lysates. Streptavidin agarose was used to adsorb specific biotin-DNA–protein complexes, which were then subjected to LC-MS/MS analysis. The pulldown results showed that, in comparison with *BLM*-G4-MUT, *BLM*-G4-WT significantly enriched 1369 kinds of proteins from cell supernatant lysates (FC > 1.5, *P*-value < 0.05, Fig. 1f). Thereinto, the top 10 regulatory protein candidates with the highest pulldown FCs (*BLM*-G4-WT versus *BLM*-G4-MUT) were chosen for subsequent RNAi validation (Fig. 1f, g; Supplementary Fig. S2c). Consequently, knockdown of STAT1, a *GAS* (gamma-activated sequences)-binding nuclear transcription factor well-known in regulating immune responses and cellular life processes [40], inhibited *BLM* gene expression significantly in both colon cancer cell lines (Fig. 1g; Supplementary Fig. S2d). G4 pulldown coupled with western blot experiments confirmed that cellular STAT1 specifically binds to *BLM*-G4-WT, but not to the *BLM*-G4-MUT sequence (Fig. 1h). Altogether, these findings highlight the crucial role of STAT1 in upregulating *BLM* transcription through its interaction with *BLM*-G4.

### Phosphorylated STAT1 exhibits a benign affinity for parallel G4s

**Figure 2.**
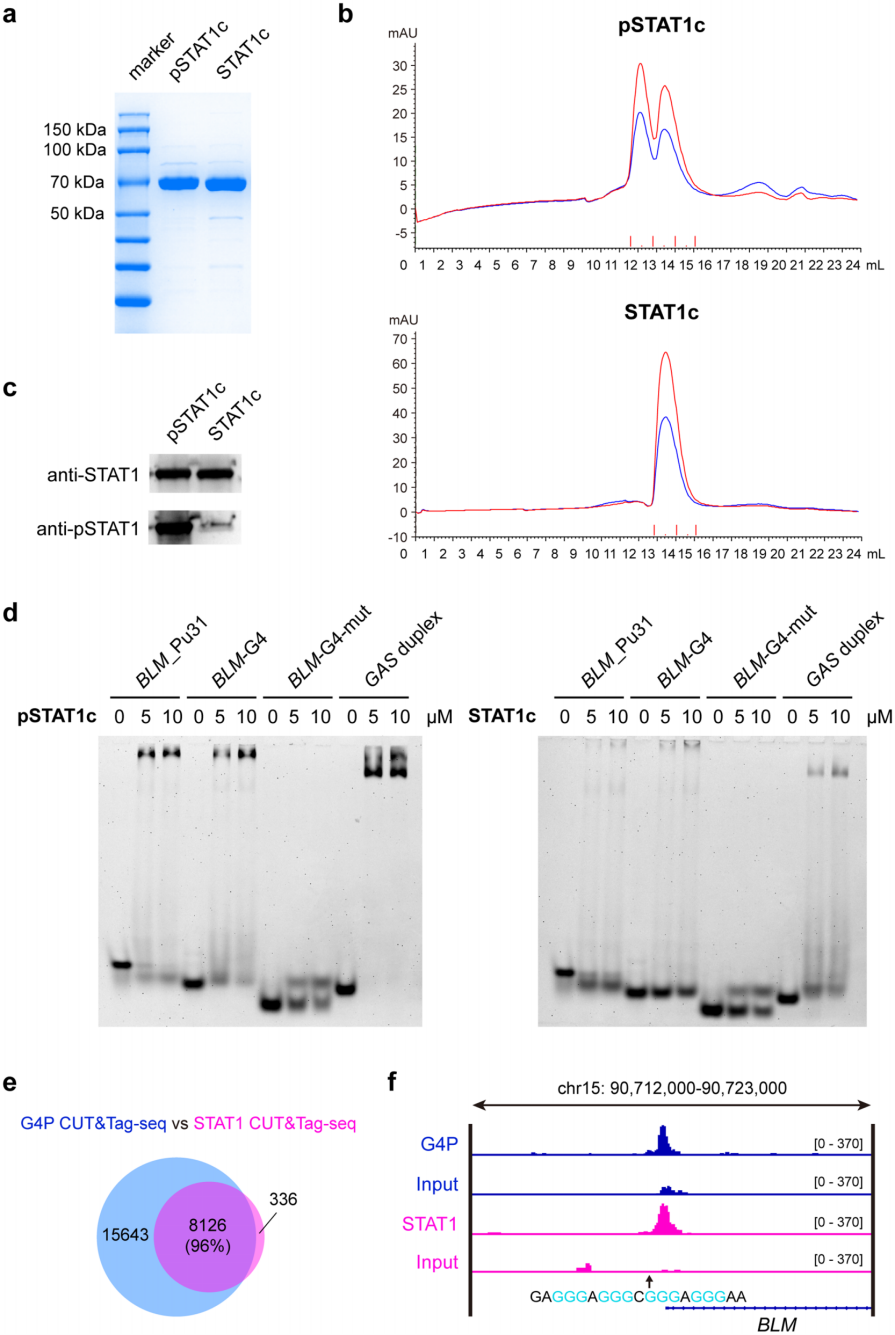
Phosphorylated STAT1 exhibits a benign affinity for parallel G4s. (**a**) SDS–PAGE results of purified pSTAT1c and STAT1c from *E. coli* TKB1 and BL21(DE3), respectively. (**b**) Size exclusion chromatography results of pSTAT1c and STAT1c. (**c**) Western blot results of purified pSTAT1c and STAT1c using anti-STAT1 and anti-phosphoSTAT1 (Tyr701) antibodies, respectively. (**d**) The EMSA results detecting the binding of pSTAT1c and STAT1c to different DNA structures. (**e**) Venn diagrams illustrating the overlap of peaks between G4-CUT&Tag-seq (GSE296647) and STAT1-CUT&Tag-seq in HCT116 cells. (**f**) Genome browser view showing that the G4 signal overlapped with the STAT1 signal in the *BLM* promoter.

To provide direct evidence for the interaction between STAT1 and *BLM*-G4, the core fragments of STAT1 without or with phosphorylation (STAT1c and pSTAT1c) were respectively expressed in the *E. coli* BL21(DE3) and *E. coli* TKB1 strain, a derivative of BL21(DE3) that carried the ELK-kinase-encoding plasmid [34]. Tyrosine kinase induction medium was used to induce tyrosine kinase expression in *E. coli* TKB1, enabling the subsequent phosphorylation of STAT1c (pSTAT1c). STAT1c and pSTAT1c were respectively extracted and purified using the His-tag Purification Resin (Fig. 2a). Size exclusion chromatography results showed that STAT1c was eluted as a monomer, whereas pSTAT1c predominantly formed a stable dimer (Fig. 2b). Western blot analysis with different antibodies confirmed the robust Tyr701 phosphorylation of pSTAT1c, while STAT1c expressed in *E. coli* BL21(DE3) showed negligible phosphorylation (Fig. 2c). To compare the DNA binding activity between STAT1c and pSTAT1c, the two proteins were respectively incubated with *BLM*_Pu31, *BLM*-G4, *BLM*-G4-mut, and the *GAS* duplex, then subjected to EMSA (Fig. 2d). The results showed that pSTAT1c could bind well to *BLM*_Pu31, *BLM*-G4, and *GAS* duplex DNA, with a clear complex band present in the lanes, but not to *BLM*-G4-mut (Fig. 2d). Notably, *BLM*_Pu31 showed higher binding affinity than *BLM*-G4, which might benefit from its natural long flanking sequences by providing more physical interactions with the protein. In contrast, unphosphorylated STAT1c showed negligible binding to any of these DNA substrates, suggesting that the phosphorylation of STAT1 is strictly required for its DNA binding activity. Further competitive EMSA experiments demonstrated that pSTAT1c specifically recognized parallel G4s, such as *KRAS*-G4 and *MYC*-G4, whereas hybrid G4s (*Tel-hybrid-1* and *Tel-hybrid-2*), antiparallel-G4 (*BOM-17*), and random ssDNA structures failed to bind to pSTAT1c (Supplementary Fig. S3). Overall, these data demonstrated that pSTAT1 is a newly discovered parallel G4-interacting protein.

To investigate the G4-binding broadness of STAT1 at the genome-wide level, STAT1-CUT&Tag-seq was conducted on HCT116 cells. The peak overlap analysis between STAT1-CUT&Tag-seq and recently published G4-CUT&Tag-seq (GSE296647) [41] showed that 96% of STAT1 peaks were colocalized with G4P peaks across the genome of HCT116 cells (Fig. 2e), strongly supporting STAT1 as a broadly G4-associated transcription factor. Meanwhile, the exact colocalization of G4P and STAT1 signals at the *BLM* promoter region provides direct evidence that STAT1 binds to the endogenous *BLM* promoter G4 to upregulate the expression of *BLM* (Fig. 2f).

### *BLM* promoter G4 is strongly bound by berberine/coptisine

**Figure 3.**
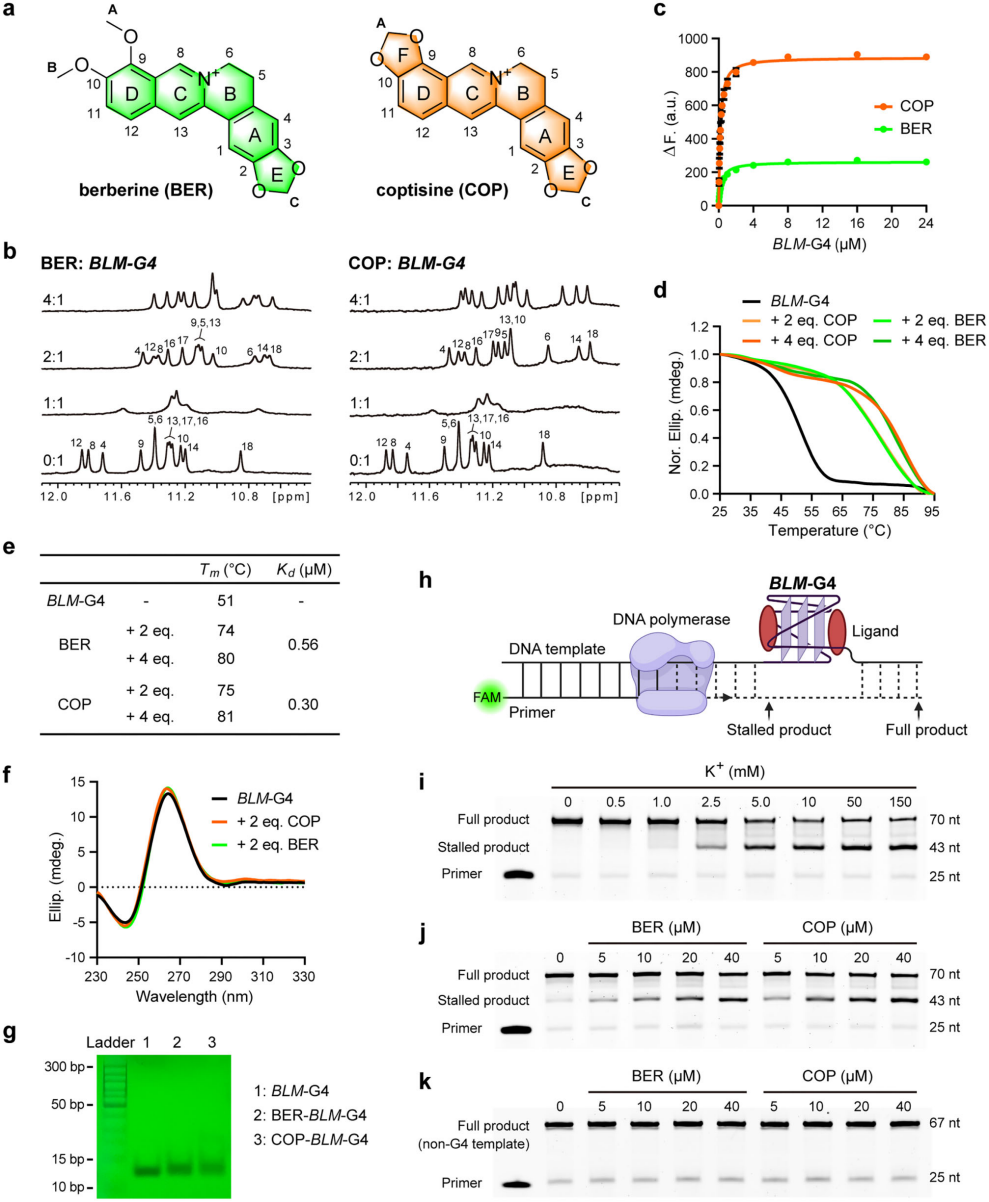
*BLM* promoter G4 is strongly bound by BER/COP. (**a**) The chemical structures of BER and COP. (**b**) ^1^H-NMR titration spectra of *BLM*-G4 with BER and COP, respectively. Conditions: 150 μM DNA, pH 7.0, 50 mM K^+^ solution, 25°C. (**c**) Determination of the *K*_d_ of BER and COP for *BLM*-G4 by the fluorescence titration assay. Conditions: 0.2 μM BER/COP, pH 7.0, 50 mM K^+^ solution, 25°C. Statistical data are shown as the mean ± SD of three independent experiments. (**d**) The CD melting curves of *BLM*-G4 and its complex with BER or COP, determined at 264 nm. Conditions: 20 μM DNA, pH 7.0, 0.1 mM K^+^ solution. (**e**) The *K*_d_ and *T*_m_ values of BER and COP for *BLM*-G4 based on (c) and (d), respectively. (**f**) The CD spectra of *BLM*-G4 and its complex with BER or COP. Conditions: 20 μM DNA, pH 7.0, 0.1 mM K^+^ solution, 25°C. (**g**) The EMSA result of *BLM*-G4 (*BLM*_Pu21T), and its complexes with 4 equivalents of BER and COP. Conditions: 150 μM DNA, pH 7.0, 50 mM K^+^ solution, 25°C. (**h**) The schematic of the DPSA. Created in BioRender. Wang, Y. (2026) https://BioRender.com/d95w663. (**i**) *BLM*-G4 stalled the progression of DNA polymerase as the K^+^ concentration increased. (**j**) BER and COP could bind and stabilize *BLM*-G4, which further inhibited the progression of DNA polymerase as the drug concentration increased (1 mM K^+^ condition). (**k**) BER and COP had no inhibitory effect on the replication of non-G4 DNA template (1 mM K^+^ condition).

BER and COP (Fig. 3a) are canonical G4-binding crescent-shaped alkaloids, which have shown strong binding affinity for many oncogenic promoter G4s, partly accounting for their anti-tumor activity [42–44]. To determine whether BER and COP could bind to the parallel *BLM*-G4, a ^1^H-NMR titration assay was conducted. The ^1^H-NMR titration results showed that BER and COP could bind well to *BLM*-G4, as evidenced by the notable up-field chemical shifts of 12 imino protons at 10–12 ppm (Fig. 3b). According to the peak numbers and chemical shift changes, the binding stoichiometry between BER/COP and *BLM*-G4 was determined as 2:1. The fluorescence titration assay revealed micromolar binding affinity of BER and COP for *BLM*-G4, with *K*_d_ values of 0.56 and 0.30 μM, respectively (Fig. 3c, e). Meanwhile, the binding of BER or COP greatly enhanced the thermal stability of *BLM*-G4 (Δ*T*_m_: 23/24°C at 2 equivalents of BER/COP) without influencing its monomeric parallel topology (Fig. 3d–g). These results suggest a possible function for BER and COP in regulating *BLM* expression by binding and stabilizing *BLM*-G4.

To further verify the stabilization ability of BER/COP on *BLM*-G4, a DPSA was conducted (Fig. 3h). The results showed that the formation of *BLM*-G4 within the long promoter sequence context effectively impeded DNA polymerase progression under gradient K^+^ concentrations (Fig. 3i). Moreover, at a low K^+^ concentration, BER and COP dose-dependently reinforced the elongation interception (Fig. 3j), while the two compounds had no impact on non-G4 template-based replication (Fig. 3k). These results further confirmed the strong binding and stabilization effect of BER/COP on *BLM*-G4.

### Berberine/coptisine suppresses *BLM* expression by binding to *BLM*-G4 in competition with STAT1

**Figure 4.**
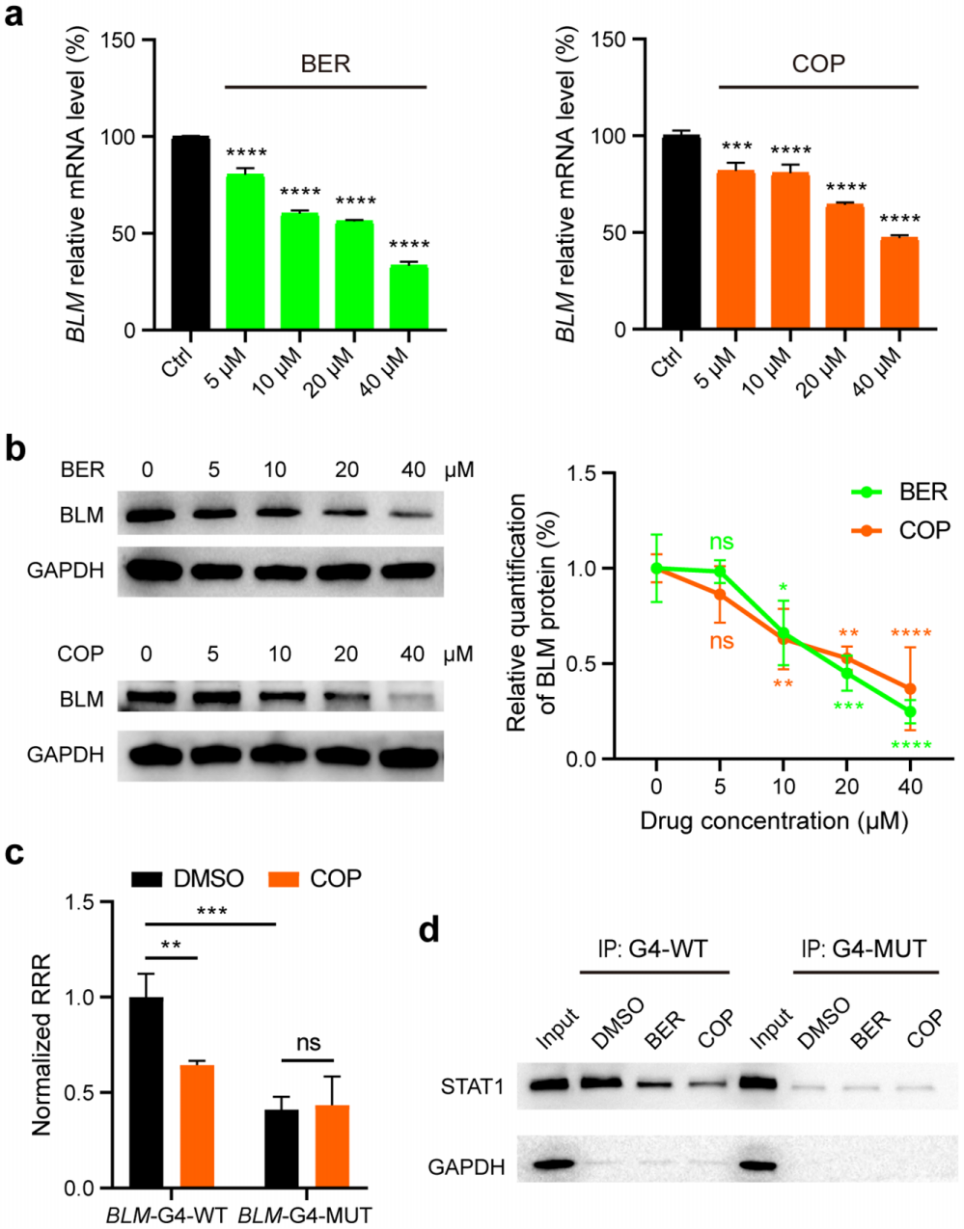
BER/COP suppresses *BLM* expression by binding to *BLM*-G4 competitively with STAT1. (**a**) The inhibition of *BLM* transcription in HT-29 cells under 48 h treatment with BER and COP, respectively. (**b**) Western blot and quantification results of BLM protein levels in HT-29 cells under 48 h treatment with BER or COP. (**c**) Dual-luciferase reporter assay results of HT-29 cells transfected with *BLM*-G4-WT/MUT plasmids after 36 h treatment with 40 μM COP. RRR (relative response ratio) = (Renilla response)/(Firefly response). (**d**) STAT1 protein in HT-29 cells was specifically pulled down by *BLM*-G4-WT, which was competed out when *BLM*-G4-WT was pre-incubated with BER/COP. Statistical data are shown as means ± SD of three independent experiments. *P-*values (ns, not significant; **P < 0.01; ***P < 0.001; ****P < 0.0001) were determined by one-way ANOVA with post-hoc Dunnett’s test or two-way ANOVA with post-hoc Sidak’s test.

To characterize the potential effect of BER/COP on *BLM* gene expression by binding to *BLM*-G4, cellular experiments were conducted on the two colon cancer cell lines (HCT116 and HT-29) with high BLM expression. Firstly, CCK-8 results showed that BER and COP induced the death of HCT116 and HT-29 cells dose-dependently (Supplementary Fig. S4). Subsequently, qPCR and western blot experiments demonstrated that both BER and COP could significantly decrease the mRNA and protein levels of *BLM* (Fig. 4a, b; Supplementary Fig. S5a–c). These findings revealed that BER and COP treatment could significantly inhibit *BLM* gene expression and promote the death of colon cancer cells. To identify whether the downregulation of *BLM* mediated by BER/COP involved the *BLM* promoter G4, a drug-based dual-luciferase reporter assay was conducted on the two cancer cell lines treated with COP (Fig. 4c; Supplementary Fig. S5d). The results showed that COP significantly inhibited the activity of Renilla luciferase launched by the *BLM*-G4-WT promoter, while it exhibited no effect on the *BLM*-G4-MUT group, indicating that COP inhibited the expression of the downstream gene by binding to the *BLM* promoter G4. To determine whether BER/COP-mediated *BLM* downregulation involves the functional absence of STAT1, which had been verified as a positive regulator of *BLM* gene expression by binding to *BLM*-G4 (Figs 1 and 2; Supplementary Fig. S2d), a *BLM*-G4 pulldown coupled with western blot experiment was conducted utilizing colon cancer cell lysates. The results showed that, upon incubation with BER/COP, the ability of *BLM-*G4 in pulling down the STAT1 protein from HT-29/HCT116 cell lysates was greatly reduced (Fig. 4d; Supplementary Fig. S5e), which revealed the competitive binding of *BLM*-G4 between BER/COP and STAT1. *In vitro* EMSA results confirmed again that *BLM*-G4–BER/COP complexes inhibited the binding of pSTAT1c to *BLM*-G4, as seen from the faded complex bands of *BLM*-G4-pSTAT1c with gradient concentrations of BER/COP (Supplementary Fig. S5f). Altogether, these results indicated that BER and COP could bind to *BLM*-G4 in competition with STAT1 in the *BLM* promoter region, thereby inhibiting *BLM* gene expression in colon cancer cells.

### Solution structure determination of *BLM*-G4 and its complexes with berberine/coptisine

**Figure 5.**
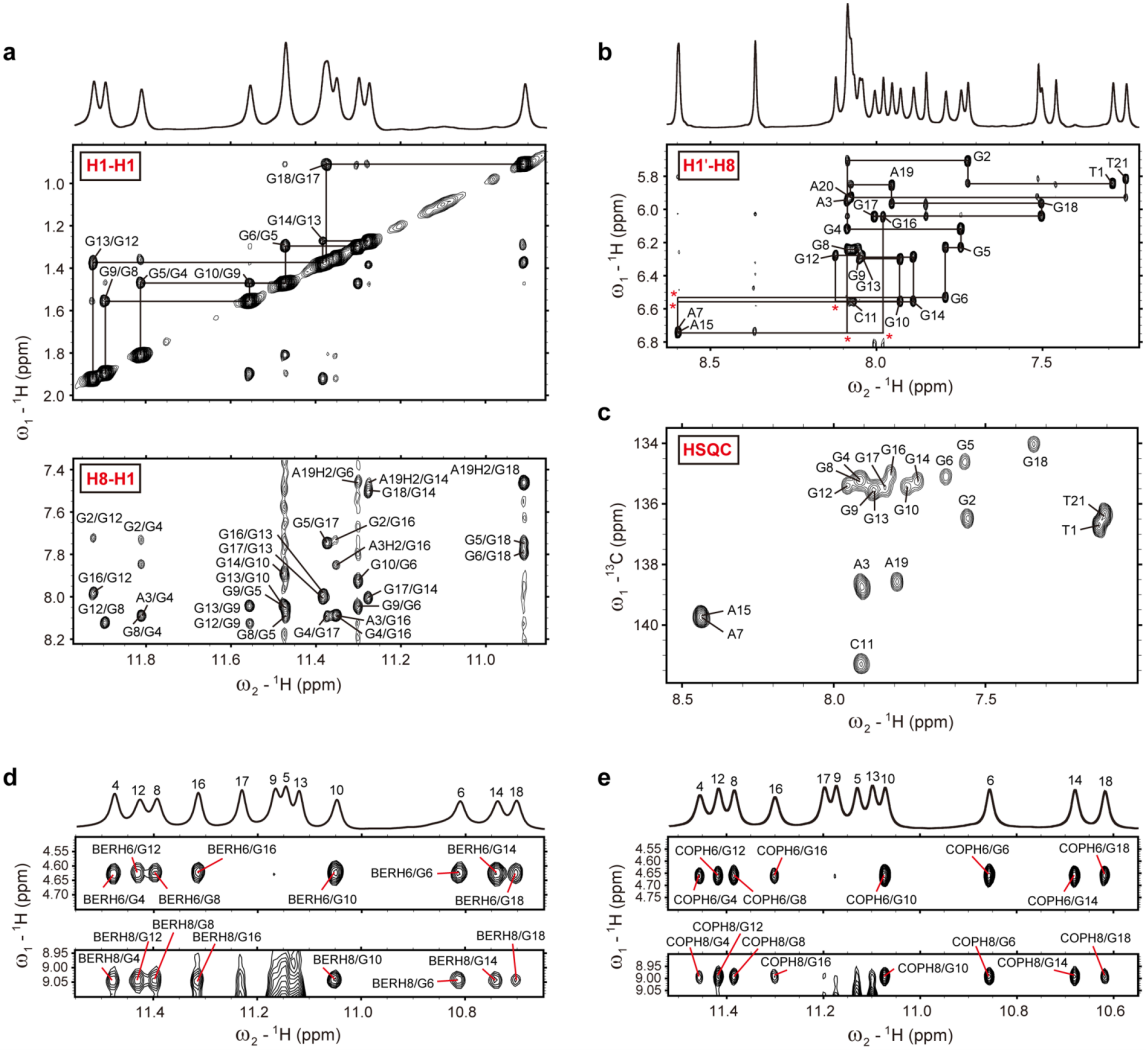
The signal assignments of free *BLM*-G4 and BER/COP–*BLM*-G4 complexes in the NMR spectra. (**a** and **b**) The signal ssignments of H1–H1 and H8–H1 regions (**a**) as well as the H1′–H8 region (**b**) in the NOESY spectrum of free *BLM*-G4. Conditions: 1.62 mM *BLM*-G4, pH 7.0, 50 mM K^+^, 15°C. (**c**) C8−H8/C6−H6 cross-peak assignments in the HSQC spectrum of free *BLM*-G4. Conditions: 1.93 mM *BLM*-G4, pH 7.0, 50 mM K^+^, 25°C. (**d** and **e**) The intermolecular H6–H1 and H8–H1 correlation signals of BER–*BLM*-G4 (**d**) and COP–*BLM*-G4 (**e**) in their NOESY spectra. Conditions: ligand:*BLM*-G4 ratio = 2:1, pH 7.0, 50 mM K^+^, 25°C.

**Figure 6.**
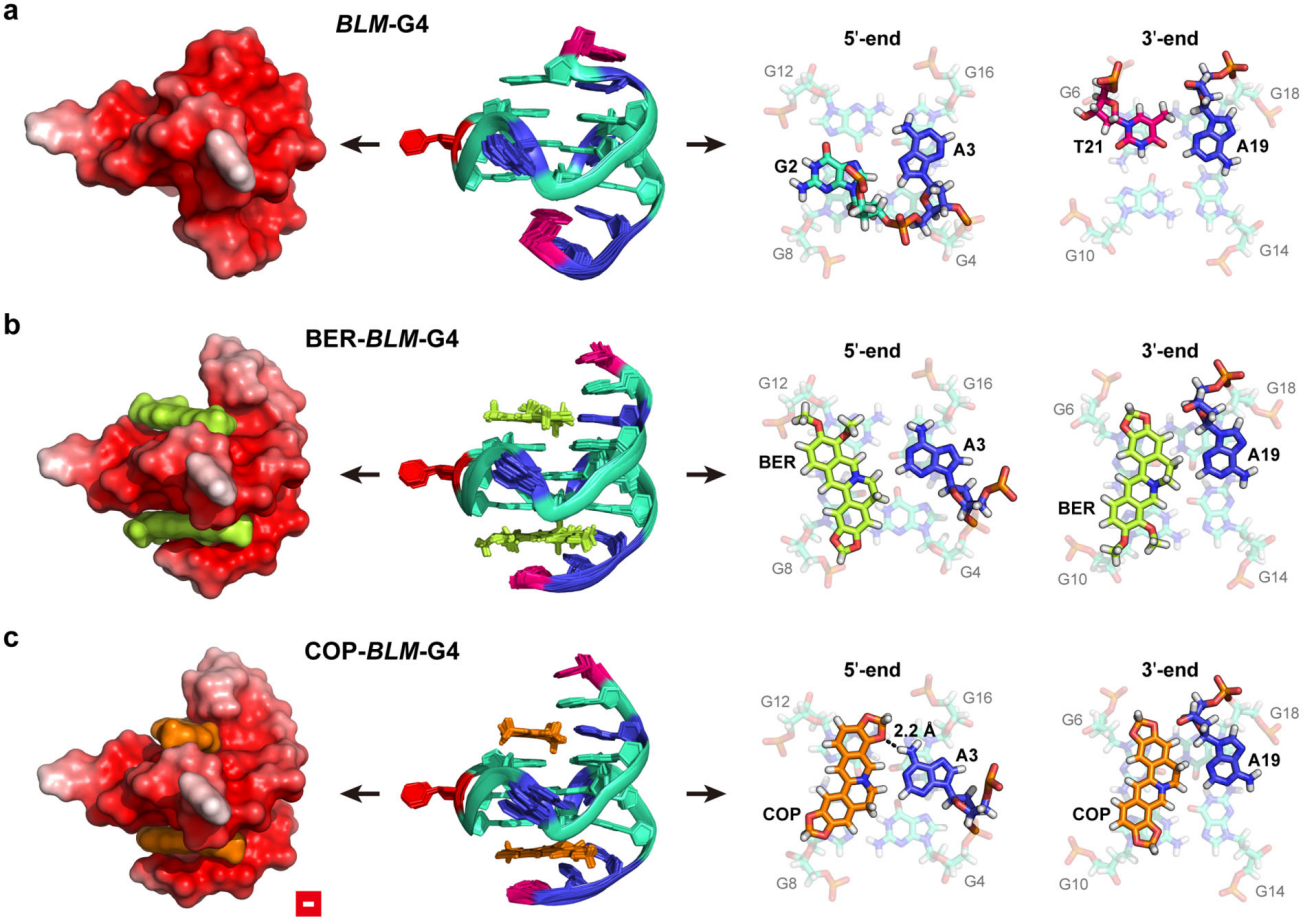
The 10 overlapped lowest-energy solution structures (middle), APBS electrostatic calculation results (left, for the lowest-energy structure), and the 5′- and 3′-end flanking structures (right, for the lowest-energy structure) of *BLM*-G4 (**a**) and its complex with BER (**b**) or COP (**c**), respectively.

To gain structural insights into *BLM*-G4 and its binding complexes with BER/COP at the atomic levels, we determined their solution structures *in vitro* by using restrained molecular dynamics simulations incorporated with 2D NOESY information. Firstly, the assigned signals from NOESY, HSQC, and DQF-COSY spectra (Fig. 5; Supplementary Tables S4–S18; Supplementary Figs S6, S7, and S9–S15) highlighted the constant three-layer G-tetrad composition of *BLM*-G4 in both free and ligand-bound modes, i.e. G4–G8–G12–G16, G5–G9–G13–G17, and G6–G10–G14–G18, as depicted in the *BLM*-G4 model (Fig. 1c, left). Then, the D_2_O exchange experiment validated the accuracy of G-residue assignments as the last four remaining imino proton peaks denoted the middle G-tetrad (Supplementary Fig. S8). Subsequently, utilizing NOE-restrained molecular dynamic simulations, we obtained the 10 lowest-energy solution structures of free *BLM*-G4 and its complexes with the ligands BER and COP (Fig. 6; Supplementary Fig. S16). These structures displayed good convergence with a heavy atom root-mean-square deviation (RMSD) of 0.51 ± 0.10, 0.58 ± 0.20, and 0.54 ± 0.12 Å (all residues) for free *BLM*-G4, BER–*BLM*-G4, and COP–*BLM*-G4, respectively (Supplementary Table S19).

Specifically, the free *BLM*-G4 structure exhibited that a G2:A3 two-base co-plane was formed at the 5′-end, with T1 steadily stacked onto A3 through a π-stacking interaction; whereas for the 3′-end flanking residues, A20 was stacked beneath A19, with T21 folded back, and formed a co-plane with A19 (Fig. 6a; Supplementary Fig. S17a). In contrast, the single-nucleotide propeller loops A7, C11, and A15 were rather stretched into solution. After the addition of BER or COP, significant rearrangements of the 5′- and 3′-end flanking structures were observed, while the conformation of propeller loops remained unchanged (Fig. 6; Supplementary Figs S16 and S17). Due to the structural similarity between the two ligands and the accessibility of *BLM*-G4’s binding pocket, BER and COP shared common characteristics while binding to *BLM*-G4, including the recruitment of the same flanking residues to form ligand–base co-planes at both G4 ends and their similar stacking onto the same G residues (Fig. 6b, c). In addition, the nitrogen cations of BER and COP were all attracted to the center of the outer G tetrads due to the inner electron-rich cavity of *BLM*-G4. For COP, in particular, its O3 site formed a 2.2 Å hydrogen bond with A3H61 at the 5′-end of *BLM*-G4, which makes COP deflected clockwise relative to BER at this binding pocket (Fig. 6c). The hydrogen bond formation also enhanced the binding force of COP with *BLM*-G4, as evidenced by its *T*_m_ and *K*_d_ discrepancy with BER–*BLM*-G4 (Fig. 3e). Overall, as seen in the binding modes of BER/COP with *BLM*-G4 (Fig. 6b, c; Supplementary Fig. S18), it is denoted that the CA, CC, C1, C12, and C13 sites of BER and COP (Fig. 3a) could be modified by introducing side chains or conjugated with base analogs, duplex DNA minor groove binders, or oligonucleotides [45] to enhance the affinity and selectivity towards *BLM*-G4.

### Coptisine produces a general G4 stabilization effect in colon cancer cells

**Figure 7.**
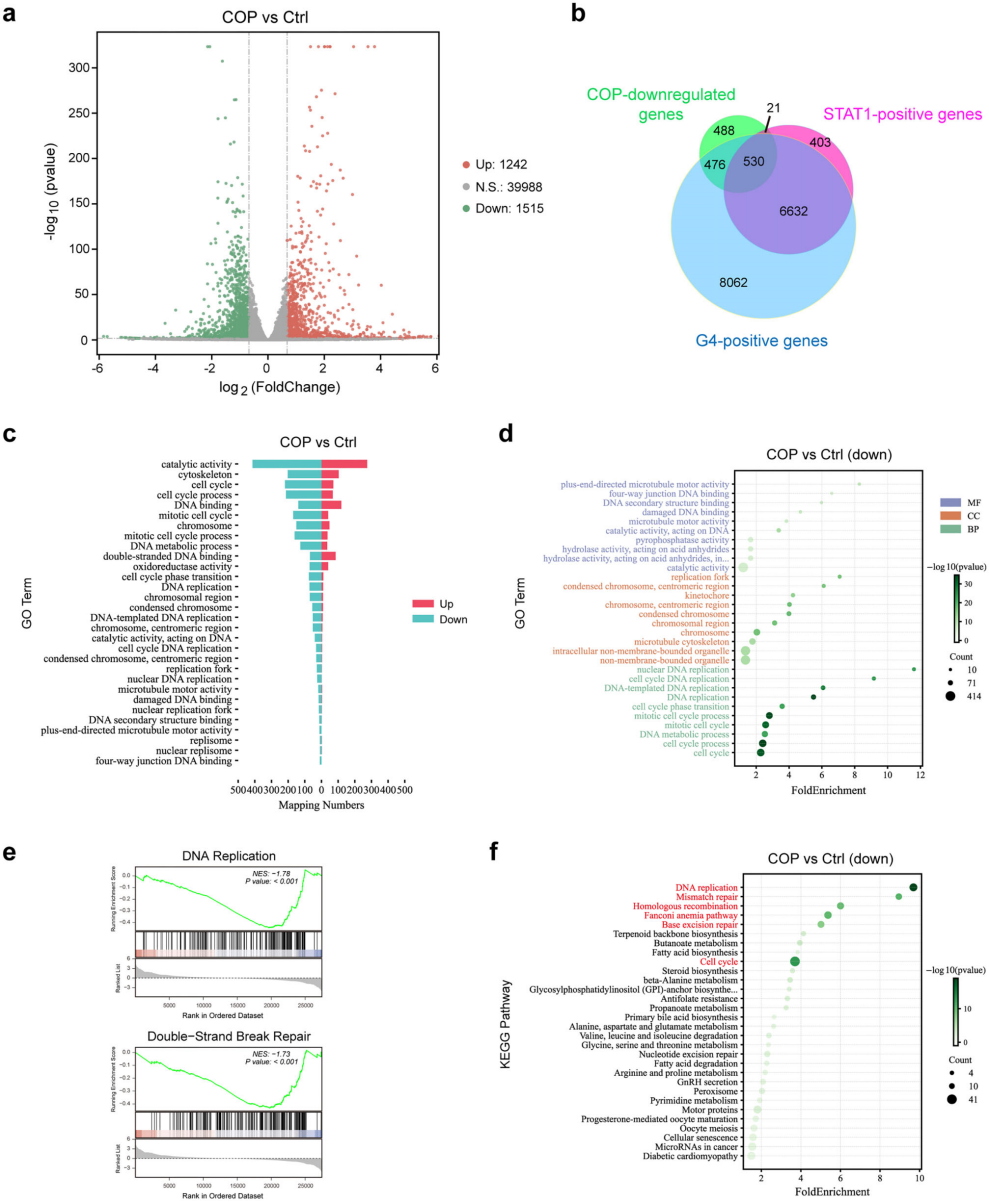
COP produces a general G4 stabilization effect in colon cancer cells. (**a**) Volcano plot of upregulated and downregulated genes in HCT116 cells after 24 h treatment with 60 μM COP, determined by RNA-seq. (**b**) Venn diagrams illustrating the overlap between COP-downregulated genes, STAT1-positive genes, and G4-positive genes in HCT116 cells. (**c**) Butterfly bar chart illustrating the significantly regulated terms in HCT116 cells after COP treatment by GO enrichment analysis. (**d**) GO enrichment analysis of the top 10 downregulated terms in Cellular component (CC), Molecular functions (MF), and Biological process (BP) from COP-treated HCT116 cells. (**e**) GSEA of significantly regulated terms associated with DNA replication and DSB repair in COP-treated HCT116 cells. (**f**) KEGG enrichment analysis of the top 30 downregulated pathways in COP-treated HCT116 cells.

Since BER and COP had been reported as extensive G4-binding alkaloids [42], we conducted an RNA-seq experiment on HCT116 cells treated with COP. The RNA-seq volcano plot showed that 1242 genes were significantly upregulated while 1515 genes (including *BLM*) were prominently downregulated (|log2(FC)| > 0.678, *P*-value < 0.05) (Fig. 7a). The overlap analysis between COP-RNA-seq, G4-CUT&Tag-seq (GSE296647), and STAT1-CUT&Tag-seq revealed that two-thirds of downregulated genes mediated by COP treatment carried G4s (Fig. 7b). Additionally, >96% of STAT1-positive genes downregulated by COP were G4-colocalized (Fig. 7b). These statistical results indicated COP as a general G4–STAT1 intervener to regulate gene expression in colon cancer cells. The Gene Ontology (GO) term analysis of significantly regulated genes showed that many of them were associated with DNA binding, DNA replication, DNA metabolic process, chromosomes, and DNA repair (Fig. 7c). Within the genes downregulated by COP treatment, their correlation with genetic information processing was much higher, as presented by GO and Kyoto Encyclopedia of Genes and Genomes (KEGG) enrichment analysis in Fig. 7d and f. Gene set enrichment analysis (GSEA) results exhibited two examples that DNA replication and DSB repair terms were mainly enriched by downregulated genes (Fig. 7e). These findings together indicate that COP mainly downregulates the G4-containing genes associated with DNA metabolism and repair (including *BLM*) to induce the death of colon cancer cells. Similarly, an RNA-seq analysis conducted by the Jie Cao group showed that BER treatment significantly downregulated genes associated with the Fanconi anemia pathway, HR, transcriptional misregulation, and other genetic metabolic pathways in HCT116 cells, thereby exacerbating colon cancer cell death [46]. Therefore, it is extrapolated that COP and BER exert similar functions in inhibiting genetic information processing-relevant pathways and genes, including *BLM*, to accelerate the death of colon cancer cells.

### Berberine/coptisine synergizes with olaparib to kill colon cancer cells

**Figure 8.**
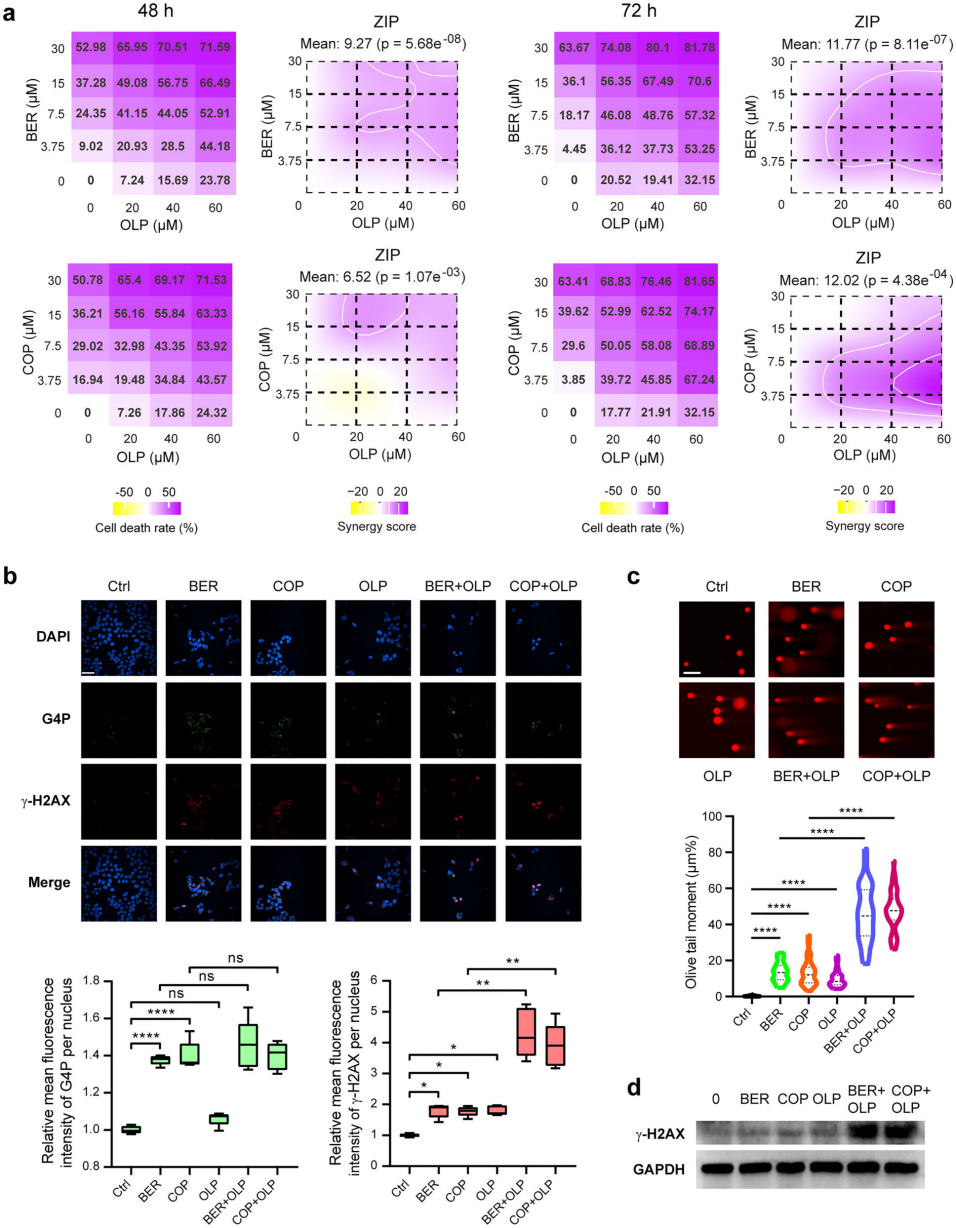
BER/COP synergizes with OLP to kill colon cancer cells. (**a**) The cell death rates and ZIP synergy plots of HT-29 cells under treatment with different drug combinations for 48 h (left) and 72 h (right), respectively. Cell death rates are shown as means of three independent experiments. (**b**) FAM-G4P and γ-H2AX immunofluorescence results in HT-29 cells under treatment with different drug combinations for 48 h. Scale bar: 50 μm. (**c**) DNA damage visualization of HT-29 cells after the treatment with different drug combinations for 48 h by the single-cell comet assay. The olive tail moments were calculated based on at least 50 cells per sample. Scale bar: 50 μm. (**d**) The western blotting results of γ-H2AX in HT-29 cells after 48 h treatment with different drug combinations. BER/COP at 15 μM and OLP at 40 μM were fixed for drug combination experiments. Statistical data are shown as means ± SD of at least three independent experiments. *P*-values (ns, not significant; *P < 0. 05; **P < 0.01; ****P < 0.0001) were determined by one-way ANOVA with post-hoc Dunnett’s test.

Drug combination has been shown as a vital strategy against complex cancers, especially those that have developed resistance to single drugs [47]. Given that COP/BER treatment significantly downregulated HR pathway genes (including *BLM*), we decided to investigate the feasibility of combining BER/COP with PARP inhibitors as an effective therapeutic strategy for colon cancer treatment. The viabilities of HT-29 and HCT116 cells under treatment with OLP were first determined (Supplementary Fig. S19a). Consequently, though both cell lines were BRCA-proficient, HCT116 cells were found to be much more sensitive to OLP than HT-29 cells, which might be due to the known mismatch repair pathway (MMR) deficiency and corresponding microsatellite instability of HCT116 cells [48]. The drug synergy evaluation based on cell viabilities showed that OLP and COP/BER were highly synergistic in HT-29 cells (synergy score > 6) under 48 and 72 h drug treatment, while they only exhibited a simple additive effect in HCT116 cells (−10 < synergy score < 0) (Fig. 8a; Supplementary Fig. S19b). Therefore, we further investigated the drug combination effects on HT-29 cells with 15 μM BER/COP and 40 μM OLP. Results from comet assay, γ-H2AX immunofluorescence, and western blotting demonstrated that the drug combination intensified DSBs greatly, thereby accelerating cancer cell death (Fig. 8). Overall, these findings underline the therapeutic potential of combining BER/COP with PARP inhibitors for treating colon cancers.

## Discussion

**Figure 9.**
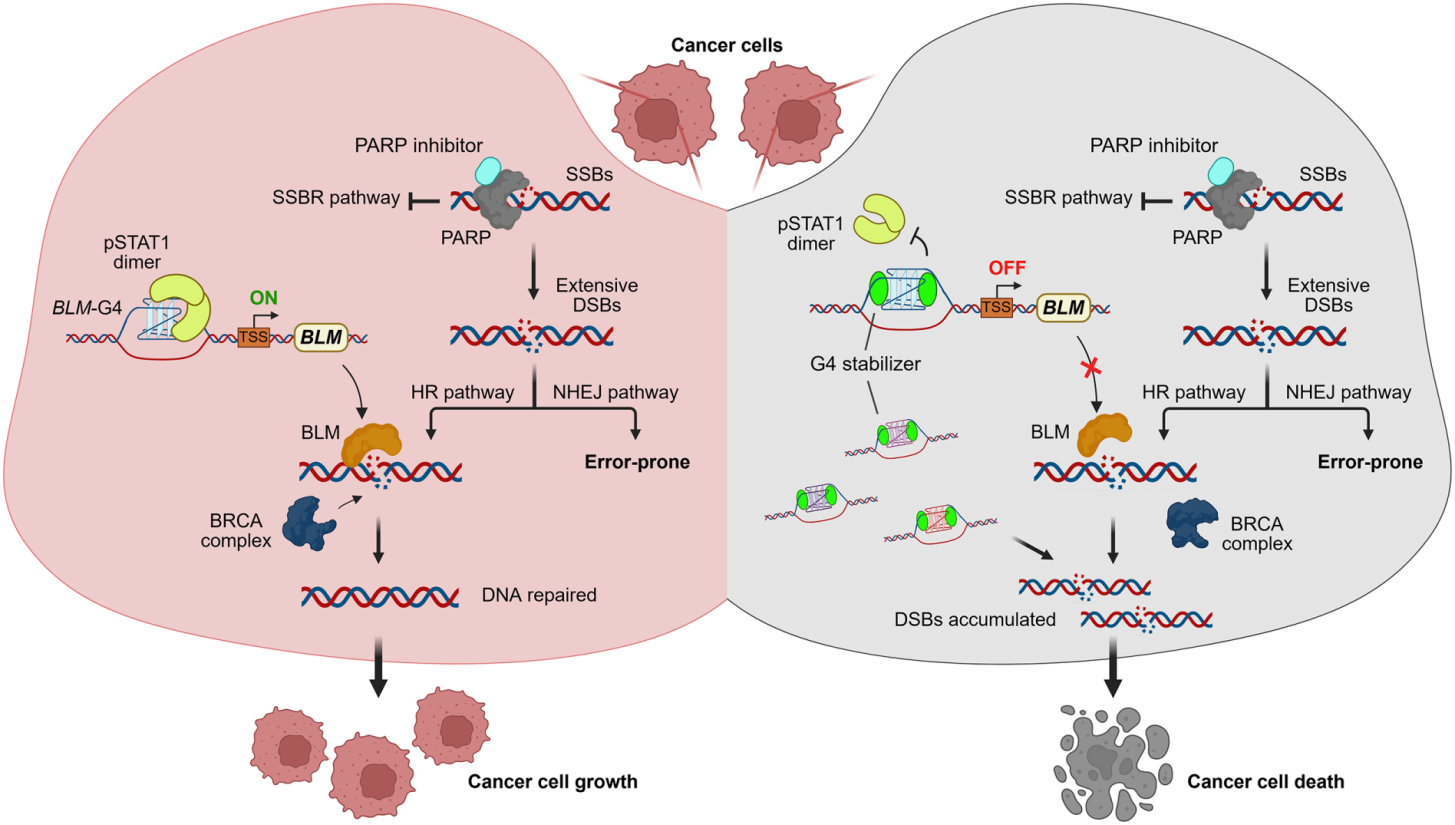
The schematic mechanism of the G4 stabilizer–PARP inhibitor drug combination in promoting cancer cell death by inducing extensive DSBs. Schematic created in BioRender. Wang, Y. (2026) https://BioRender.com/6xha898.

In summary, we report for the first time that the canonical *GAS*-binding transcriptional activator STAT1 interacts with *BLM*-G4 in the promoter to upregulate *BLM* gene expression in colon cancer cells. Furthermore, we found that two natural G4-binding alkaloids, BER and COP, significantly inhibit *BLM* expression by binding to *BLM*-G4 and displacing STAT1. To gain structural insights into the *BLM*-G4–ligand binding, we determined the NMR solution structures of free *BLM*-G4 and its complexes with BER and COP. With the binding pockets revealed, these structures provide valuable information for targeted drug discovery and optimization. Beyond *BLM* regulation, our integrated multi-omics analysis, combining genome-wide STAT1-CUT&Tag-seq, G4-CUT&Tag-seq, and COP-RNA-seq, revealed STAT1 as a broad G4-binding transcription factor and COP as a pan-genomic G4 stabilizer. COP-mediated G4 stabilization downregulates numerous G4-positive genes essential for genetic information processing, such as DNA replication, metabolism, and DNA repair. Moreover, BER and COP exhibit a remarkable synergistic effect with OLP in inducing the death of colon cancer cells by disrupting DNA repair pathways (including the *BLM* signaling pathway) and accumulating DSBs (Fig. 9). Mechanistically, PARP plays a central role in the single-strand break repair (SSBR) pathway, which is responsible for the rapid repair of single-strand breaks (SSBs). When PARP is inhibited, unrepaired SSBs that are encountered by advancing replication forks are prone to convert into DSBs [49], which are normally repaired through the high-fidelity HR and error-prone NHEJ pathways. Among the key players in HR repair, BLM helicase has been verified as a critical protein in recruiting HR pathway factors (such as RPA, RAD51, RAD54, and BRCA1/2) to DSBs and promoting accurate HR repair [3, 50]. Consequently, the simultaneous targeting of G4s (including *BLM*-G4) and PARP through a drug combination strategy can dramatically increase the erroneous repair and accumulation of DSBs, destructing the residual intrinsic order of cancer cells and leading to their demise, which thus provides a promising strategy for cancer therapy.

## Supplementary Material

gkag383_Supplemental_Files

## Data Availability

G4 structural data were deposited in the Protein Data Bank under accession codes 9JO0 https://doi.org/10.2210/pdb9jo0/pdb (free *BLM*-G4), 9JO1 https://doi.org/10.2210/pdb9jo1/pdb (BER–*BLM*-G4), and 9DQE https://doi.org/10.2210/pdb9dqe/pdb (COP–*BLM*-G4). *BLM*-G4 pulldown MS proteomics data have been deposited in the ProteomeXchange Consortium via the PRIDE repository with the dataset identifier PXD075345. STAT1 CUT&Tag-seq data have been deposited in the NCBI GEO database under accession code GSE324248. HCT116 RNA-seq data have been deposited in the NCBI GEO database under accession code GSE324253.
