# Supplementary material for: A small molecule disrupts G4–STAT1 interaction and synergizes with olaparib to drive cancer cell death
Source: Nucleic Acids Res. 2026 May 5;54(9):gkag383. doi: 10.1093/nar/gkag383 (PMC13139855; doi:10.1093/nar/gkag383)
Supplement: gkag383_Supplemental_Files [file gkag383_supplemental_files.zip › BLM-supporting information-NAR-00813-L-2026.pdf]

Supplementary Data

A small molecule disrupts G4–STAT1 interaction and synergizes with olaparib to drive cancer cell death

Yingying Wang[1†], Xuenan Zhang[2†], Yuting Bian[1†], Sihan He[1], Rongshuang Cheng[1], Jinzhu Li[1], Jonathan Dickerhoff[3], Yushuang Liu[1], Yu Zhou[1], Jinlei Bian[1], Kewei Zheng[2*], Danzhou Yang[3*], Ling-Yi Kong[1*] and Kai-Bo Wang[1*]

CONTENT

Table S1. The DNA sequences used in this study.
Table S2. The siRNA sequences used in this study.
Table S3. The qPCR primers used in this study.
Table S4. Proton chemical shifts of *BLM*-G4 at 15°C in 50 mM K^+-containing solution (pH=7.0).
Table S5. Inter-residue NOE signals of the 5′-end capping structure of *BLM*-G4.
Table S6. Inter-residue NOE signals of the 3′-end capping structure of *BLM*-G4.
Table S7. Inter-residue NOE signals of the single-nucleotide loops of *BLM*-G4.
Table S8. Proton chemical shifts of residues in BER-*BLM*-G4 complex at 25°C in 50 mM K^+-containing solution (pH=7.0).
Table S9. Intermolecular NOE signals between BER and *BLM*-G4.
Table S10. Inter-residue NOE signals of the 5′-end capping structure of BER-*BLM*-G4.
Table S11. Inter-residue NOE signals of the 3′-end capping structure of BER-*BLM*-G4.
Table S12. Inter-residue NOE signals of the single-nucleotide loops of BER-*BLM*-G4.
Table S13. Proton chemical shifts of residues in COP-*BLM*-G4 complex at 25°C in 50 mM K^+-containing solution (pH=7.0).
Table S14. Intermolecular NOE signals between COP and *BLM*-G4.
Table S15. Inter-residue NOE signals of the 5′-end capping structure of COP-*BLM*-G4.
Table S16. Inter-residue NOE signals of the 3′-end capping structure of COP-*BLM*-G4.
Table S17. Inter-residue NOE signals of the single-nucleotide loops of COP-*BLM*-G4.
Table S18. Proton chemical shifts of free ligands (1) and bound ligands at 25°C in 50 mM K^+-containing solution (pH=7.0).
Table S19. NMR restraints and structural statistics for *BLM*-G4, BER-*BLM*-G4, and COP-*BLM*-G4.
Figure S1. The biophysical property investigation of *BLM*_Pu31 and *BLM*_Pu21T.
Figure S2. STAT1 knockdown inhibits BLM expression significantly in colon cancer cells.
Figure S3. The binding activity of pSTAT1c to different DNA structures.
Figure S4. The inhibitory effect of BER and COP on the viability of different colon cancer cells.
Figure S5. BER and COP stabilize *BLM* promoter G4 and displace STAT1, thereby inhibiting BLM expression.
Figure S6. C8−H8/C6−H6 cross-peak assignments for A, G, T, and C bases (black labels), and C2-H2 cross-peak assignments for A bases (red labels) in the HSQC spectrum of free *BLM*-G4.
Figure S7. The signal assignments of free *BLM*-G4 in its DQF-COSY spectrum.
Figure S8. Imino proton spectra of free *BLM*-G4 during different time courses of the D_2O exchange experiment.
Figure S9. The full ^1H NMR spectra of *BLM*-G4 with and without coptisine (COP) or berberine (BER), respectively.
Figure S10. The signal assignments of H1-H1, H8−H1, and H1′-H8 regions from the NOESY spectrum of BER-*BLM*-G4.

Figure S11. The signal assignments of H1-H1, H8−H1, and H1′-H8 regions from the NOESY spectrum of COP-*BLM*-G4.

Figure S12. C8−H8/C6−H6 cross-peak assignments for A, G, T, and C bases (black labels), and C2-H2 cross-peak assignments for A bases (red labels) in the HSQC spectrum of BER-*BLM*-G4.

Figure S13. C8−H8/C6−H6 cross-peak assignments for A, G, T, and C bases (black labels), and C2-H2 cross-peak assignments for A bases (red labels) in the HSQC spectrum of COP-*BLM*-G4.

Figure S14. The signal assignments of BER-*BLM*-G4 in its DQF-COSY spectrum.

Figure S15. The signal assignments of COP-*BLM*-G4 in its DQF-COSY spectrum.

Figure S16. The top and bottom views of 10 overlapped lowest-energy solution structures of *BLM*-G4, BER-*BLM*-G4, and COP-*BLM*-G4, respectively.

Figure S17. The lateral structures, 5′-end and 3′-end capping structures of *BLM*-G4 (a), BER-*BLM*-G4 (b), and COP-*BLM*-G4 (c).

Figure S18. The top and bottom views of BER-*BLM*-G4 (a) and COP-*BLM*-G4 (b) in surface mode underlying the possible binding grooves of *BLM*-G4 for drug derivatization.

Figure S19. The effects of olaparib (OLP) and drug combination with BER/COP on HT-29 and HCT116 cell viabilities.

Table S1. The DNA sequences used in this study.

Name	Sequence (5′ → 3′)
*BLM*_Pu31	AAGAGGAGGGAGGGCGGGAGGGAAGGAGAGA
*BLM*_Pu21T (*BLM*-G4)	TGAGGGAGGGCGGGAGGGAAT
***BLM*-G4 pulldown assay**	
BLM-G4-WT-biotin	TTTTTTGAGGGAGGGCGGGAGGGAATTTTTT-TEG-biotin
BLM-G4-MUT-biotin	TTTTTTGAGTGAGTGCGTGAGTGAATTTTTT-TEG-biotin
**STAT1-G4 EMSA**	
BLM-G4-FAM	5′-6-FAM-TGAGGGAGGGCGGGAGGGAAT
*BLM*_Pu31-FAM	5′-6-FAM-AAGAGGAGGGAGGGCGGGAGGGAAGGAGAGA
GAS duplex-FAM	Forward: 5′-6-FAM-ACAGTTTCCCGTAAATGC;<br>Reverse: GCATTTACGGGAAACTGT
BLM-G4-mut-FAM	5′-6-FAM-TGAGTGAGTGCGTGAGTGAAT
**STAT1-G4 competitive EMSA**	
*BLM*_Pu31-FAM	5′-6-FAM-AAGAGGAGGGAGGGCGGGAGGGAAGGAGAGA
*BLM*_Pu31	AAGAGGAGGGAGGGCGGGAGGGAAGGAGAGA
BLM-G4	TGAGGGAGGGCGGGAGGGAAT
KRAS-G4	TGAGGGCGGTGTGGGAATAGGGAA
MYC-G4	TGAGGGTGGGTAGGGTGGGTAA
Tel-hybrid-1	AAAGGGTTAGGGTTAGGGTTAGGGAA
Tel-hybrid-2	TTAGGGTTAGGGTTAGGGTTAGGGTT
BOM-17	GGTTAGGTTAGGTTAGG
GAS duplex	Forward: ACAGTTTCCCGTAAATGC;<br>Reverse: GCATTTACGGGAAACTGT
BLM-G4-mut	TGAGTGAGTGCGTGAGTGAAT
**DPSA assay**	
DPSA primer	5′-6-FAM-TAATACGACTCACTATAGCAATTGC
BLM-G4 template	T**CACAAAGAGGAGGGAGGGCGGGAGGGAAGGAGAGA**T<br>AGCTGCACGCAATTGCTATAGTGAGTCGTATTA
non-G4 template	TTGCTGCCTTGTGCAGATGTGTGCGTGACCAGATAGCTGCACG<br>CAATTGCTATAGTGAGTCGTATTA

Table S2. The siRNA sequences used in this study.

siRNA	Sequence (5′ → 3′)
Control	GCGACGAUCUGCCUAAGAU
TFAM	GAGGGAACUUCCUGAUUCA
RCC1	CAGCAGCCCUCACCGAUGA
UBAP2L	CAACACAGCAGCACGUUAU
CTCF	GUAGAAGUCAGCAAAUUAA
ZFR	GCACUUAAAAGGGCGAAGA
SF1	GACCUGACUCGUAAACUGC
SNRPA1	UGGUAUAAUAGCCUUGUUU
RAD50	CUGCGACUUGCUCCAGAUAAAUU
RTRAF	GCUAAGGCAAAUCAAACAA
STAT1	GCAGGUUCACCAGCUUUAU

Table S3. The qPCR primers used in this study.

Name	Forward (5′ → 3′)	Reverse (5′ → 3′)
GAPDH	CATGAGAAGTATGACAACAGCCT	AGTCCTTCCACGATACCAAAGT
BLM	AAGTTTCCTTCTGTTCCGGTGAT	ACACCTGAGGTCTGAGAATCTTCA
TFAM	AGCTCAGAACCCAGATGCAA	CCGCCCTATAAGCATCTTGA
RCC1	GGCTTGGTGCTGACACTAGGC	CCTCCACTGATGTGTCCCTTC
UBAP2L	ACACAATCCCCATCACTGGT	CAGAGGAGAAGACGGAGGTG
CTCF	ATGTTATATTTGTCATGCTCGGTTTA	TTTCGGGCTATGACTGTGTC
ZFR	TCCCAATGCTAAGGAGATGC	TTCTTCTCGTCTTCGCCAGT
SF1	CCTTCGGGAAGACGATAACA	TTCAGCCATGAGGGACAAAT
SNRPA1	GGTGCTACGTTAGACCAGTTTG	GTCCCTCACCTATACGGCATATT
RAD50	CATGTTGCCCCGTTTGTCAG	GTCCCAGCATTTCATCACGC
RTRAF	TGTTCCGACGCAAGTTGA	CGCTGCTGTGGATGTTTCT
STAT1	ACTCAAAATTCCTGGAGCAG	ACGCTTGCTTTTCCTTATGTT
Renilla luciferase	CTGCCTCCAGCTACCTGTG	GGAAGGTTCAGCAGCTCG
Firefly luciferase	GCATCGTGGTGTGCTCTG	CTGCAGGCCCTTCTTAGAC

Table S4. Proton chemical shifts of *BLM*-G4 at 15°C in 50 mM K^+-containing solution (pH=7.0).

Base	H1/H2/H5/Me	H6/H8	H1′	H2′, H2″	H3′	H4′	H5′, H5″ *
**T1**	1.71	7.29	5.84	1.67, 2.11	4.50	3.88	3.55, 3.55
**G2**		7.72	5.70	2.56, 2.49	4.80	4.19	3.80, 3.89
**A3**	7.85	8.08	5.94	2.52, 2.65		4.13	3.76, 3.94
**G4**	11.81	8.09	6.11	2.82, 3.04	5.05	4.52	4.12, 4.18
**G5**	11.47	7.74	6.23	2.65, 2.96	5.06	4.61	4.34, 4.34
**G6**	11.30	7.79	6.53	2.79, 2.70	5.20	4.72	4.39, 4.46
**A7**	8.36	8.60	6.74	2.98, 2.98	5.29	4.78	4.37, 4.39
**G8**	11.89	8.09	6.24	2.57, 3.04	5.24	4.56	4.35, 4.46
**G9**	11.55	8.05	6.30	2.75, 3.02	5.17	4.66	4.35, 4.37
**G10**	11.47	7.93	6.55	2.81, 2.66	5.18	4.72	4.37, 4.48
**C11**	6.24	8.07	6.55	2.48, 2.80	5.17	4.71	4.37, 4.46
**G12**	11.92	8.12	6.28	2.59, 3.06	5.26	4.56	4.33, 4.44
**G13**	11.38	8.04	6.28	2.76, 3.01	5.18	4.66	4.39, 4.49
**G14**	11.28	7.89	6.55	2.79, 2.68	5.19	4.73	4.40, 4.50
**A15**	8.36	8.59	6.74	2.98, 2.98	5.29	4.78	4.37, 4.39
**G16**	11.35	7.98	6.04	2.41, 2.84	5.19	4.53	4.36, 4.45
**G17**	11.37	8.00	6.04	2.72, 2.80	5.12	4.59	4.23, 4.32
**G18**	10.91	7.50	5.96	2.40, 2.75	5.02	4.53	4.22, 4.33
**A19**	7.46	7.95	5.85	2.29, 2.53		4.36	4.11, 4.21
**A20**	7.51	8.07	5.92	2.53, 2.52	4.87	4.19	4.07, 3.95
**T21**	1.56	7.25	5.81	2.09, 2.17	4.40	3.93	3.95, 4.07

Note: *Assignments are not stereospecific.

Table S5. Inter-residue NOE signals of the 5′-end capping structure of *BLM*-G4.

	G2	A3	
**T1**	**H8**	**H2**	**H8**
**H1′**	W		OL
**H2′**	M		M
**H2″**	S		M
**H3′**	M		
**H6**	W		W
**Me**		M	VW

	A3	G4	G8	G12	G16
**G2**	**H8**	**H1**	**H1**	**H1**	**H1**
**H1′**	M	W	W		
**H2′**	M	M			
**H2″**	M	M			
**H3′**	M	W			
**H4′**					
**H5′**	W				
**H5″**	W				
**H8**	M	W		W	W

	G4		G16	
**A3**	**H1**	**H8**	**H1**	**H1′**
**H1′**		OL	M	
**H2′**			VW	
**H2″**			VW	
**H2**			W	W
**H8**	W		M	

Note: S = strong intensity, M = medium intensity, W = weak intensity, VW = very weak intensity, OL = overlapped.

Table S6. Inter-residue NOE signals of the 3′-end capping structure of *BLM*-G4.

	G6	G14	G18							A20	T21
**A19**	**H1**	**H1**	**H1′**	**H2′**	**H2″**	**H3′**	**H4′**	**H1**	**H8**	**H8**	**Me**
**H1′**										M	
**H2′**										M	
**H2″**										S	
**H2**	W	W						M			VW
**H8**			M	M	S	M	W		M	W	

	T21				
**A20**	**H1′**	**H2′**	**H2″**	**H6**	**Me**
**H1′**				M	M
**H3′**				W	
**H4′**				M	M
**H5′**				OL	W
**H5″**				OL	W
**H2**	W	VW	VW		M
**H8**				W	S

	G6			
**T21**	**H2′**	**H2″**	**H1**	**H8**
**H1′**			VW	
**H6**	W	W		
**Me**				W

Note: S = strong intensity, M = medium intensity, W = weak intensity, VW = very weak intensity, OL = overlapped.

Table S7. Inter-residue NOE signals of the single-nucleotide loops of *BLM*-G4.

	G6			G8	G9
**A7**	**H1′**	**H3′**	**H4′**	**H3′**	**H8**
**H1′**			W	W	
**H3′**	W				
**H4′**					W
**H8**		W	M		

	G12
**C11**	**H8**
**H3′**	W
**H4′**	W

	G14		G16		G17
**A15**	**H1′**	**H4′**	**H3′**	**H8**	**H8**
**H1′**			W		
**H3′**	W				
**H4′**				VW	VW
**H8**		M			

Note: M = medium intensity, W = weak intensity, VW = very weak intensity.

Table S8. Proton chemical shifts of residues in BER-*BLM*-G4 complex at 25°C in 50 mM K^+-containing solution (pH=7.0).

Base	H1/H2/H5/HMe	H6/H8	H1′	H2′, H2″	H3′	H4′	H5′, H5″ *
**T1**	1.52	7.08	5.65	1.41, 1.85	4.28	3.66	3.31, 3.32
**G2**		7.37	5.44	2.20, 2.12	4.59	3.88	3.49, 3.61
**A3**	7.78	8.06	6.06	2.54, 2.61	4.83	4.10	3.81, 3.87
**G4**	11.35	7.95	5.93	2.63, 2.87	4.89	4.37	4.05, 4.09
**G5**	11.03	7.54	6.03	2.64, 2.64	4.89	4.44	4.18, 4.26
**G6**	10.69	7.60	6.29	2.66, 2.56	5.06	4.53	4.19, 4.26
**A7**	8.22	8.46	6.57	2.82, 2.81	5.11	4.58	4.20, 4.28
**G8**	11.28	7.86	6.01	2.32, 2.82	5.04	4.39	4.19, 4.28
**G9**	11.05	7.72	6.03	2.64, 2.64	4.96	4.45	4.17, 4.27
**G10**	10.93	7.64	6.32	2.68, 2.55	5.03	4.51	4.20, 4.26
**C11**	6.10	7.93	6.39	2.31, 2.63	4.98	4.50	4.18, 4.27
**G12**	11.31	7.81	6.01	2.32, 2.82	5.02	4.38	4.17, 4.26
**G13**	11.00	7.73	6.06	2.52, 2.82	4.98	4.46	4.18, 4.27
**G14**	10.62	7.72	6.36	2.70, 2.57	5.06	4.55	4.21, 4.25
**A15**	8.22	8.46	6.58	2.83, 2.81	5.12	4.59	4.20, 4.28
**G16**	11.19	7.78	5.91	2.27, 2.70	5.04	4.39	4.19, 4.28
**G17**	11.11	7.77	5.89	2.57, 2.58	4.97	4.42	4.09, 4.18
**G18**	10.58	7.32	5.98	2.06, 2.30	4.90	4.37	4.10, 4.17
**A19**	7.40	8.10	5.89	2.47, 2.52	4.86	4.29	3.98, 4.06
**A20**	7.39	7.99	5.77	2.37, 2.38	4.71	4.04	3.94, 3.94
**T21**	1.39	7.15	5.76	1.94, 1.96	4.26	3.74	3.78, 3.84

Note: *Assignments are not stereospecific.

Table S9. Intermolecular NOE signals between BER and *BLM*-G4.

5′-end BER

	G4	G8			G12			G16	A3
**BER**	**H1**	**H1′**	**H1**	**H8**	**H1′**	**H1**	**H8**	**H1**	**H2**
**C6**	M		W			W		M	M
**CA**					W		VW		W
**CB**					VW		M		
**CC**		W		M					
**H1**				M					
**H8**	W		W			W		W	W

3′-end BER

	G6		G10					G14		G18	A19	A20	T21	
**BER**	**H2′**	**H1**	**H1′**	**H2′**	**H2″**	**H1**	**H8**	**H1**	**H8**	**H1**	**H2**	**H2**	**H1′**	**H4′**
**C6**		M				M		M		M	M			
**CA**			VW				VW						VW	
**CB**			W	M	M		M							
**CC**	M													
**H8**		W				M		W		VW		W		
**H13**														M

Note: M = medium intensity, W = weak intensity, VW = very weak intensity.

Table S10. Inter-residue NOE signals of the 5′-end capping structure of BER-*BLM*-G4.

	G2		
**T1**	**H5′**	**H5″**	**H8**
**H1′**	M	W	W
**H2′**			M
**H2″**	M	M	S
**H3′**			M
**H4′**			VW
**H5′**			VW
**H5″**			VW
**H6**			W

	A3				
**G2**	**H1′**	**H5′**	**H5″**	**H2**	**H8**
**H1′**	W			W	M
**H2′**					M
**H2″**					S
**H3′**					M
**H8**		VW	VW	W	W

	G4		G12	G16
**A3**	**H1**	**H8**	**H1**	**H1**
**H1′**		M		W
**H2′**		M		VW
**H2″**		S		W
**H3′**		W		
**H4′**		OL		
**H2**	VW		W	W
**H8**				VW

Note: S = strong intensity, M = medium intensity, W = weak intensity, VW = very weak intensity, OL = overlapped.

Table S11. Inter-residue NOE signals of the 3′-end capping structure of BER-*BLM*-G4.

	G18					A20			
**A19**	**H1′**	**H2′**	**H2″**	**H3′**	**H8**	**H3′**	**H5′**	**H5″**	**H8**
**H1′**						VW	W	W	M
**H2′**									M
**H2″**									M
**H3′**									M
**H4′**	M								W
**H5′**	M				VW				
**H5″**	M				VW				
**H2**	VW								
**H8**	W	M	M	M	W				M

	T21				
**A20**	**H1′**	**H5′**	**H5″**	**H6**	**Me**
**H1′**		W	W	M	M
**H2′**				M	
**H2″**				M	
**H3′**				M	
**H4′**				VW	
**H2**	W				
**H8**				W	M

Note: M = medium intensity, W = weak intensity, VW = very weak intensity.

Table S12. Inter-residue NOE signals of the single-nucleotide loops of BER-*BLM*-G4.

	G6			G8		
**A7**	**H1′**	**H3′**	**H4′**	**H3′**	**H4′**	**H8**
**H1′**				W	VW	
**H3′**	W					
**H4′**	W					VW
**H8**		OL	W			

	G12	
**C11**	**H3′**	**H8**
**H1′**	W	
**H3′**		W
**H4′**		W

	G14			G16	
**A15**	**H1′**	**H3′**	**H4′**	**H3′**	**H8**
**H1′**				W	
**H3′**	W				VW
**H4′**	W				W
**H8**		OL	M		

Note: M = medium intensity, W = weak intensity, VW = very weak intensity, OL = overlapped.

Table S13. Proton chemical shifts of residues in COP-*BLM*-G4 complex at 25°C in 50 mM K^+-containing solution (pH=7.0).

Base	H1/H2/H5/HMe	H6/H8	H1′	H2′, H2″	H3′	H4′	H5′, H5″ *
**T1**	1.65	7.23	5.77	1.59, 2.02	4.44	3.81	3.47, 3.47
**G2**		7.53	5.57	2.31, 2.25	4.74	4.05	3.65, 3.76
**A3**	7.92	8.23	6.27	2.75, 2.78	5.00	4.26	4.03, 4.04
**G4**	11.46	8.10	6.07	2.77, 3.00	5.03	4.53	4.20, 4.22
**G5**	11.14	7.70	6.17	2.63, 2.94	5.05	4.58	4.33, 4.32
**G6**	10.86	7.77	6.44	2.79, 2.68	5.19	4.68	4.40, 4.35
**A7**	8.37	8.60	6.72	2.96, 2.96	5.26	4.73	4.34, 4.36
**G8**	11.39	7.99	6.16	2.47, 2.95	5.18	4.54	4.35, 4.43
**G9**	11.17	7.86	6.18	2.64, 2.94	5.11	4.60	4.31, 4.42
**G10**	11.07	7.86	6.48	2.82, 2.68	5.18	4.67	4.35, 4.42
**C11**	6.23	8.07	6.54	2.46, 2.78	5.13	4.65	4.42, 4.34
**G12**	11.42	7.99	6.17	2.46, 2.97	5.19	4.54	4.32, 4.41
**G13**	11.10	7.88	6.19	2.68, 2.96	5.13	4.61	4.32, 4.42
**G14**	10.68	7.88	6.50	2.83, 2.69	5.20	4.70	4.36, 4.40
**A15**	8.37	8.60	6.72	2.97, 2.97	5.27	4.74	4.34, 4.36
**G16**	11.30	7.93	6.03	2.39, 2.84	5.18	4.54	4.35, 4.43
**G17**	11.20	7.91	6.03	2.72, 2.73	5.13	4.58	4.32, 4.24
**G18**	10.62	7.45	6.14	2.16, 2.37	5.05	4.51	4.31, 4.26
**A19**	7.44	8.26	6.04	2.64, 2.68	5.01	4.46	4.11, 4.21
**A20**	7.54	8.16	5.96	2.56, 2.56	4.88	4.22	4.10, 4.10
**T21**	1.56	7.32	5.94	2.11, 2.12	4.42	3.92	4.02, 3.95

Note: *Assignments are not stereospecific.

Table S14. Intermolecular NOE signals between COP and *BLM*-G4.

5′-end COP

	G4	G8				G12		G16		A3
**COP**	**H1**	**H1′**	**H4′**	**H1**	**H8**	**H1**	**H8**	**H1**	**H8**	**H2**
**C6**	M			M		M		W		M
**CA**		M	W		M					
**CC**									W	
**H4**	W			W						
**H8**	W			W		M		W		W
**H11**							VW			
**H12**							M			
**H51**										M

3′-end COP

	G6				G10			G14	G18	A19		A20		
**COP**	**H2′**	**H2″**	**H1**	**H8**	**H1′**	**H1**	**H8**	**H1**	**H1**	**H4′**	**H2**	**H1′**	**H4′**	**H2**
**C6**			M			M		M	W		M			
**CA**	M	M		M						M			M	
**CC**					M									
**H4**			W						M					VW
**H8**			M			M		M	W		W			
**H11**							M							
**H12**							M							
**H52**											M	W		W

Note: M = medium intensity, W = weak intensity, VW = very weak intensity.

Table S15. Inter-residue NOE signals of the 5′-end capping structure of COP-*BLM*-G4.

	G2			
**T1**	**H4′**	**H5′**	**H5″**	**H8**
**H1′**	W	M	M	W
**H2′**				M
**H2″**		M	M	S
**H3′**				W
**H4′**				VW
**H5′**				VW
**H5″**				VW

	A3		
**G2**	**H1′**	**H2**	**H8**
**H1′**	VW	M	W
**H2′**			M
**H2″**			S
**H3′**			M
**H8**		W	W

	G4					G16
**A3**	**H4′**	**H5′**	**H5″**	**H1**	**H8**	**H1**
**H1′**	W	W	W		M	M
**H2′**					M	VW
**H2″**					S	VW
**H3′**					M	
**H4′**					M	
**H2**				W		M
**H8**						VW

Note: S = strong intensity, M = medium intensity, W = weak intensity, VW = very weak intensity.

Table S16. Inter-residue NOE signals of the 3′-end capping structure of COP-*BLM*-G4.

	G18						A20			
**A19**	**H1′**	**H2′**	**H2″**	**H3′**	**H4′**	**H8**	**H1′**	**H3′**	**H2**	**H8**
**H1′**								VW		M
**H2′**										M
**H2″**										S
**H3′**										W
**H4′**										W
**H5′**	M					VW				
**H5″**	M					VW				
**H2**							W		M	
**H8**	W	M	S	M	VW	W				M

	T21				
**A20**	**H1′**	**H5′**	**H5″**	**H6**	**Me**
**H1′**		W	W	M	W
**H2′**				M	M
**H2″**				M	M
**H3′**				M	
**H4′**				VW	
**H2**	W				
**H8**				W	M

Note: S = strong intensity, M = medium intensity, W = weak intensity, VW = very weak intensity.

Table S17. Inter-residue NOE signals of the single-nucleotide loops of COP-*BLM*-G4.

	G6			G8		
**A7**	**H1′**	**H3′**	**H4′**	**H3′**	**H4′**	**H8**
**H1′**				W	VW	
**H3′**	W					VW
**H4′**	W					VW
**H8**		W	W			

	G10	G12	
**C11**	**H4′**	**H3′**	**H8**
**H1′**		W	
**H3′**			W
**H6**	W		

	G14			G16	
**A15**	**H1′**	**H3′**	**H4′**	**H3′**	**H8**
**H1′**				W	
**H3′**	W				VW
**H4′**	W				
**H8**		W	W		

Note: W = weak intensity, VW = very weak intensity.

Table S18. Proton chemical shifts of free ligands (1) and bound ligands at 25°C in 50 mM K^+-containing solution (pH=7.0).

Proton	Free BER	Bound BER	Free COP	Bound COP
**H51**	3.26	2.56	3.26	2.76
**H52**	3.26	2.68	3.26	2.76
**H61**	4.85	4.50	4.84	4.66
**H62**	4.85	4.50	4.84	4.66
**HA**	4.13	3.63	6.13	5.81
**HB**	4.14	3.75	-	-
**HC**	6.13	5.70	6.46	6.16
**H1**	7.55	6.56	7.57	6.55
**H4**	6.99	5.95	7.00	6.17
**H8**	9.67	8.93	9.62	9.00
**H11**	8.08	7.41	7.86	7.27
**H12**	7.99	7.19	7.82	7.15
**H13**	8.55	7.80	8.59	7.92

Table S19. NMR restraints and structural statistics for *BLM*-G4, BER-*BLM*-G4, and COP-*BLM*-G4.

	BLM-G4	BER-*BLM*-G4	COP-*BLM*-G4
**NOE-Based Distance Restraints**			
Total	432	484	460
Intra-residue	273	276	259
Inter-residue			
Sequential	94	127	125
Long-range	65	40	33
Ligand-G4	-	41	43
**Other Restraints**			
Hydrogen bonds	48	48	48
Torsion angles	21	21	21
G-tetrad planarity	48	48	48
**Structural Statistics**			
**Pairwise heavy atom RMSD (Å)**			
G-tetrad core	0.39 ± 0.10	0.53 ± 0.22	0.54 ± 0.15
All residues	0.51 ± 0.10	0.58 ± 0.20	0.54 ± 0.12
**Restraint violations (Å)**			
Max. NOE	0.09	0.12	0.10
Mean NOE	0.0010 ± 0.0068	0.0005 ± 0.0054	0.0004 ± 0.0042

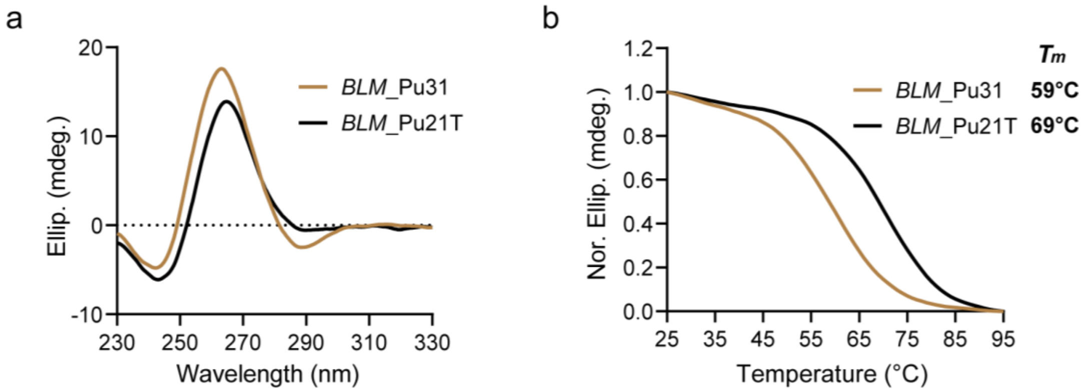

Figure S1. The biophysical property investigation of *BLM*_Pu31 and *BLM*_Pu21T. (**a**) The CD spectra of *BLM*_Pu31 and *BLM*_Pu21T. Conditions: 20 μM DNA, pH 7.0, 10 mM K^+ solution, 25°C. (**b**) The CD melting curves of *BLM*_Pu31 and *BLM*_Pu21T determined at 264 nm. Conditions: 20 μM DNA, pH 7.0, 10 mM K^+ solution.

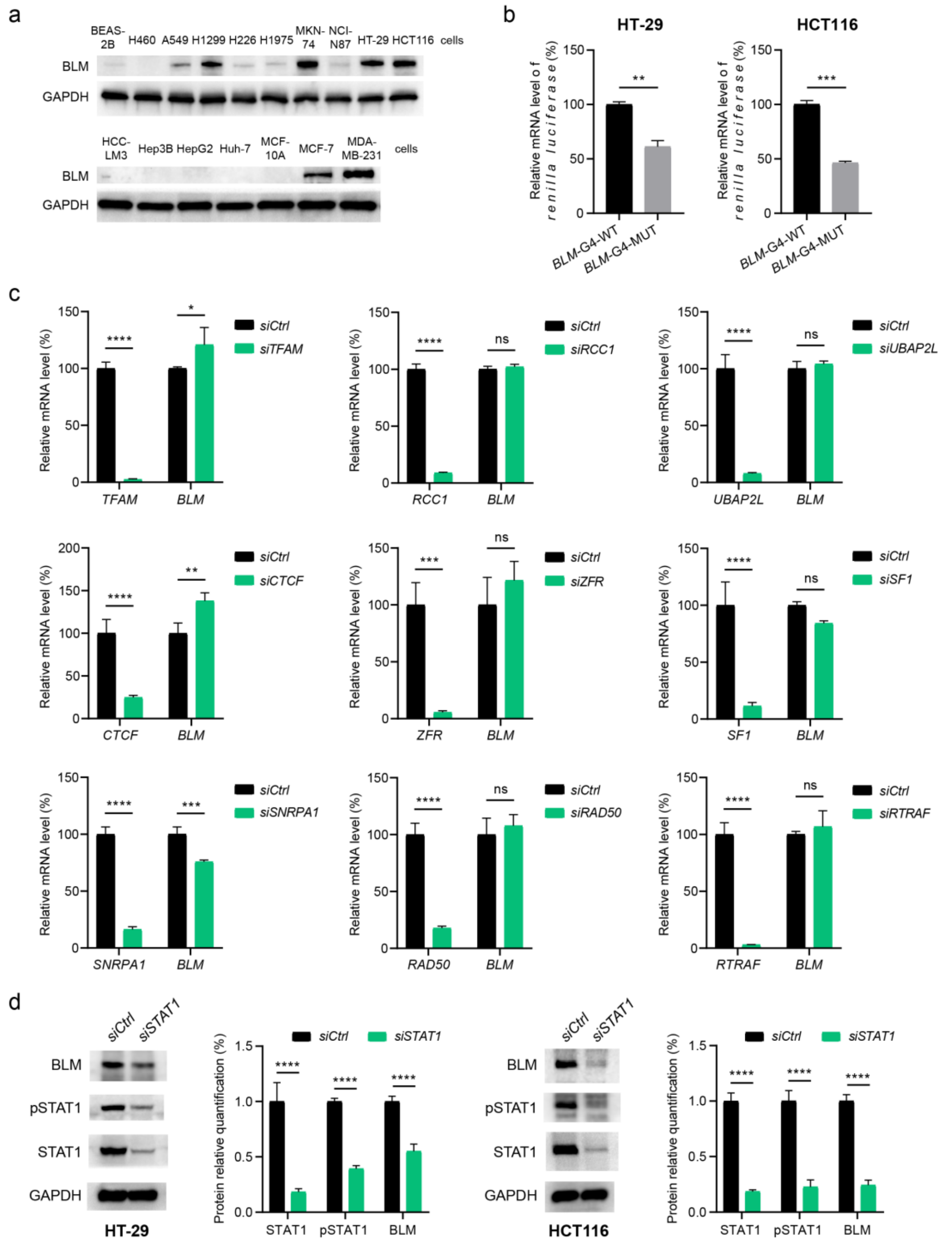

Figure S2. STAT1 knockdown inhibits BLM expression significantly in colon cancer cells. (**a**) The WB results of BLM protein expression in different cancer cells and normal cells. (**b**) Relative transcription levels of *Renilla luciferase* in HT-29 and HCT116 cells after 36 h transfection of *BLM*-G4-WT/MUT plasmids, respectively, with *Firefly luciferase* mRNA as the internal reference. (**c**) Relative transcription levels of *BLM* after knocking down different regulatory proteins for 48 h in HT-29 cells. (**d**) Detection of the protein levels of BLM and pSTAT1 after knocking down STAT1 for 60 h in HT-29 and HCT116 cells, respectively. Statistical data were shown as means ± standard deviation of three independent experiments. *P* values (ns, not significant; *, $P < 0.05$; **, $P < 0.01$; ***, $P < 0.001$; ****, $P < 0.0001$) were determined by one-way ANOVA with post hoc Dunnett.

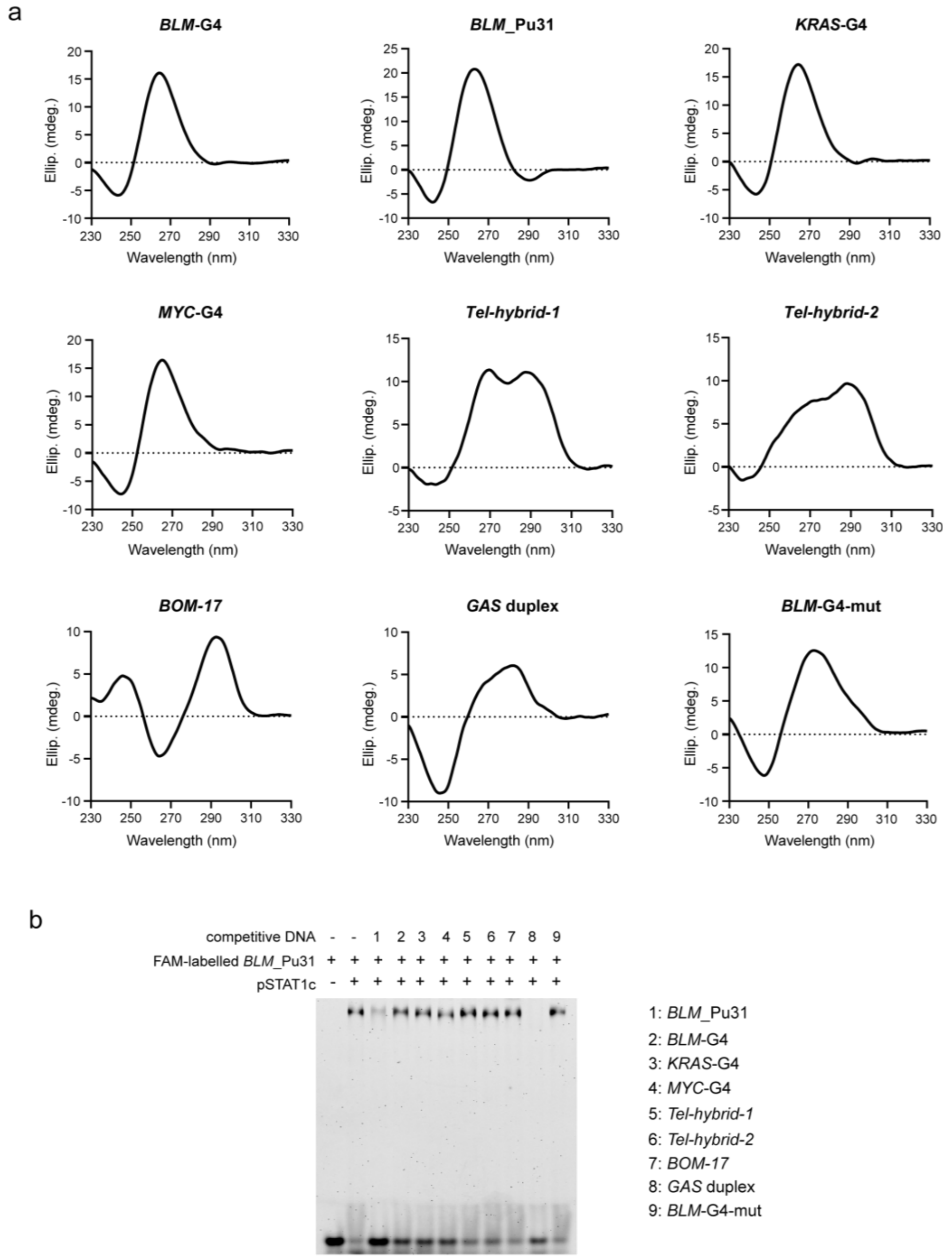

Figure S3. The binding activity of pSTAT1c to different DNA structures. (**a**) The CD spectra of different DNA structures. Conditions: 20 µM DNA, pH 7.0, 50 mM K^+ solution, 25°C. (**b**) The competitive EMSA results of pSTAT1c (5 µM) incubated with FAM-labeled *BLM*_Pu31 (0.1 µM) plus different competitive DNA (5 µM).

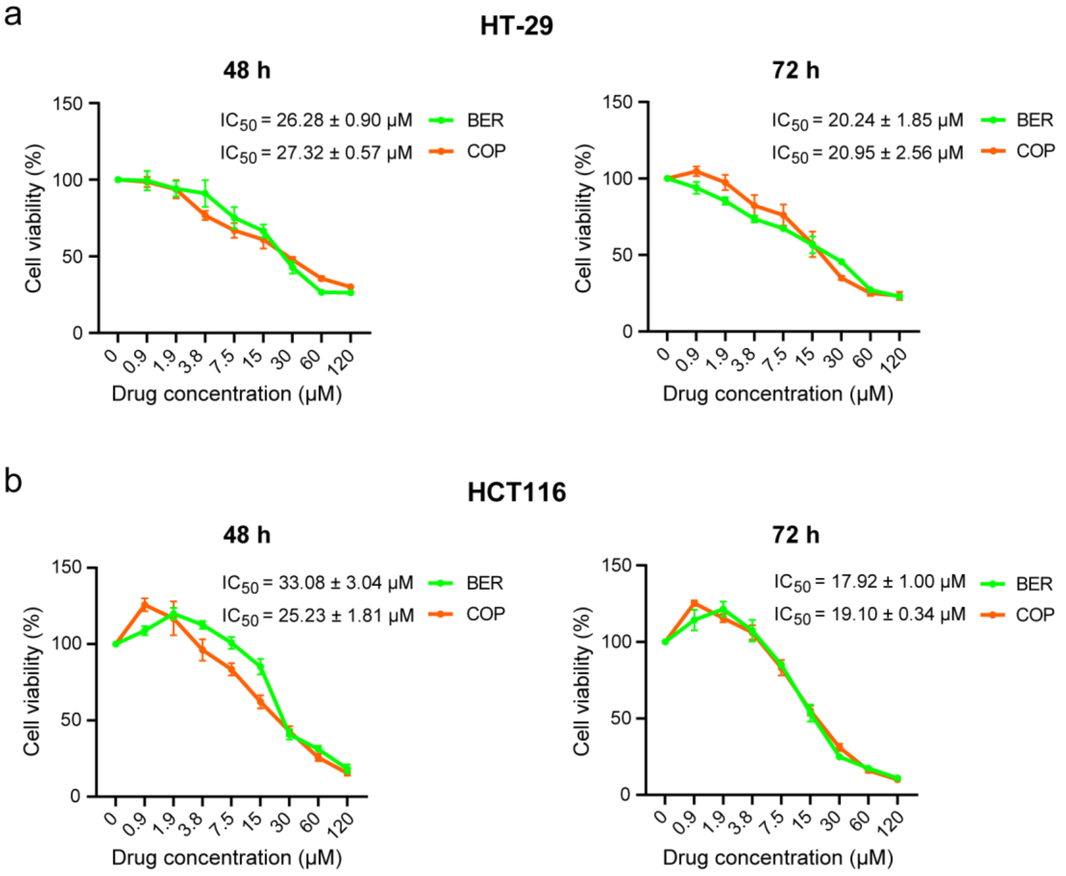

Figure S4. The inhibitory effect of BER and COP on the viability of different colon cancer cells. (**a**) HT-29 cell viability under the treatment with BER or COP for 48 and 72 h, respectively, as determined by CCK-8. (**b**) HCT116 cell viability under the treatment with BER or COP for 48 and 72 h, respectively, as determined by CCK-8. Statistical data were shown as means ± standard deviation of at least three independent experiments.

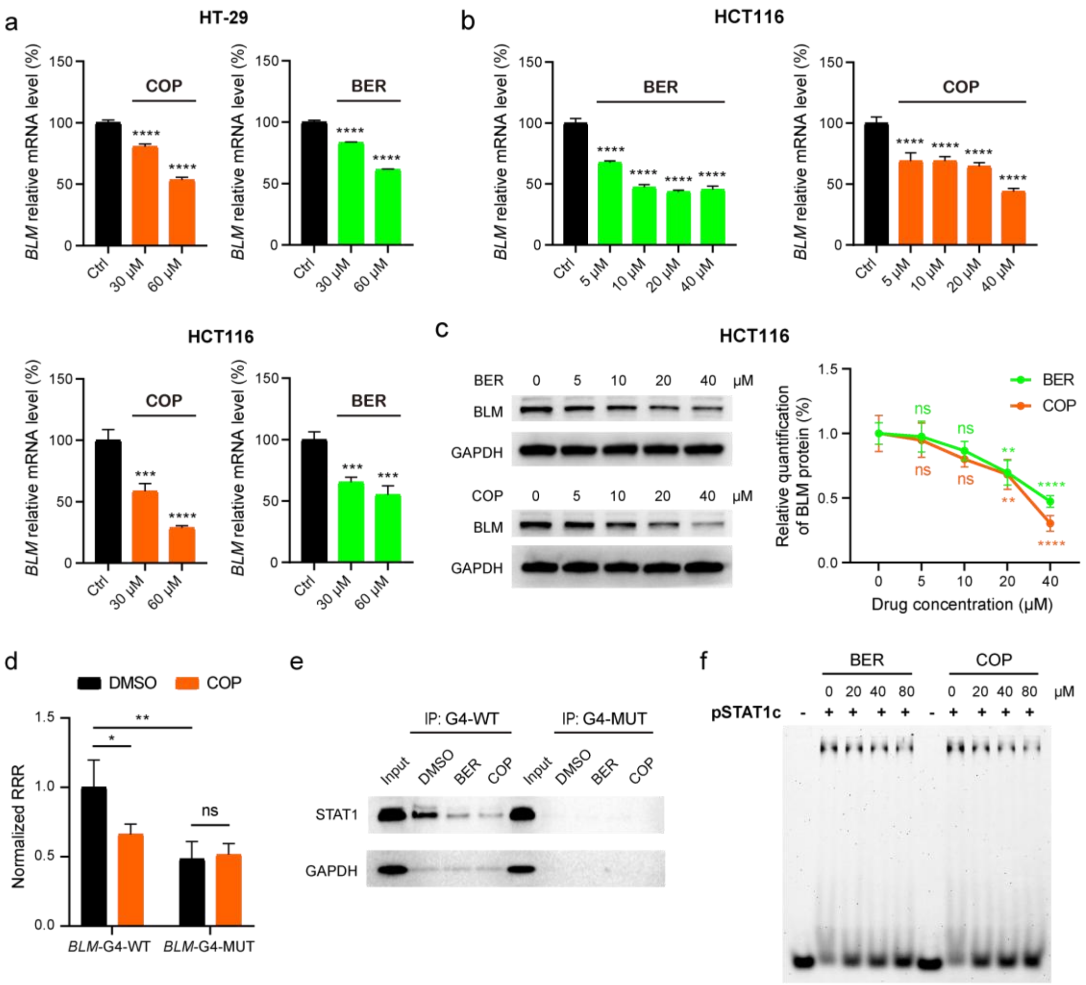

Figure S5. BER and COP stabilize *BLM* promoter G4 and displace STAT1, thereby inhibiting BLM expression. (**a**) The transcriptional changes of *BLM* in HT-29 and HCT116 cells under the treatment with BER or COP for 24 h, respectively. (**b**) The transcriptional changes of *BLM* in HCT116 cells under the treatment with BER or COP for 48 h. (**c**) Western blot and quantification results of BLM protein in HCT116 cells under the treatment with BER or COP for 48 h. (**d**) Dual luciferase reporter assay results of HCT116 cells transfected with *BLM*-G4-WT/MUT plasmids after 36 h treatment of 40 μM COP. RRR (relative response ratio) = (Renilla response)/(Firefly response). (**e**) STAT1 protein in HCT116 cells was specifically pulled down by *BLM*-G4-WT, which was competed out when *BLM*-G4-WT was precubated with BER/COP. The DMSO group was set as the control. (**f**) The EMSA results of pSTAT1c (5 μM) incubated with FAM-labeled *BLM*-G4 (0.1 μM) which was preincubated with different concentrations of BER or COP. Statistical data were shown as means ± standard deviation of three independent experiments. *P* values (ns, not significant; *, $P < 0.05$; **, $P < 0.01$; ***, $P < 0.001$; ****, $P < 0.0001$) were determined by one-way ANOVA with post hoc Dunnett or two-way ANOVA with post hoc Sidak.

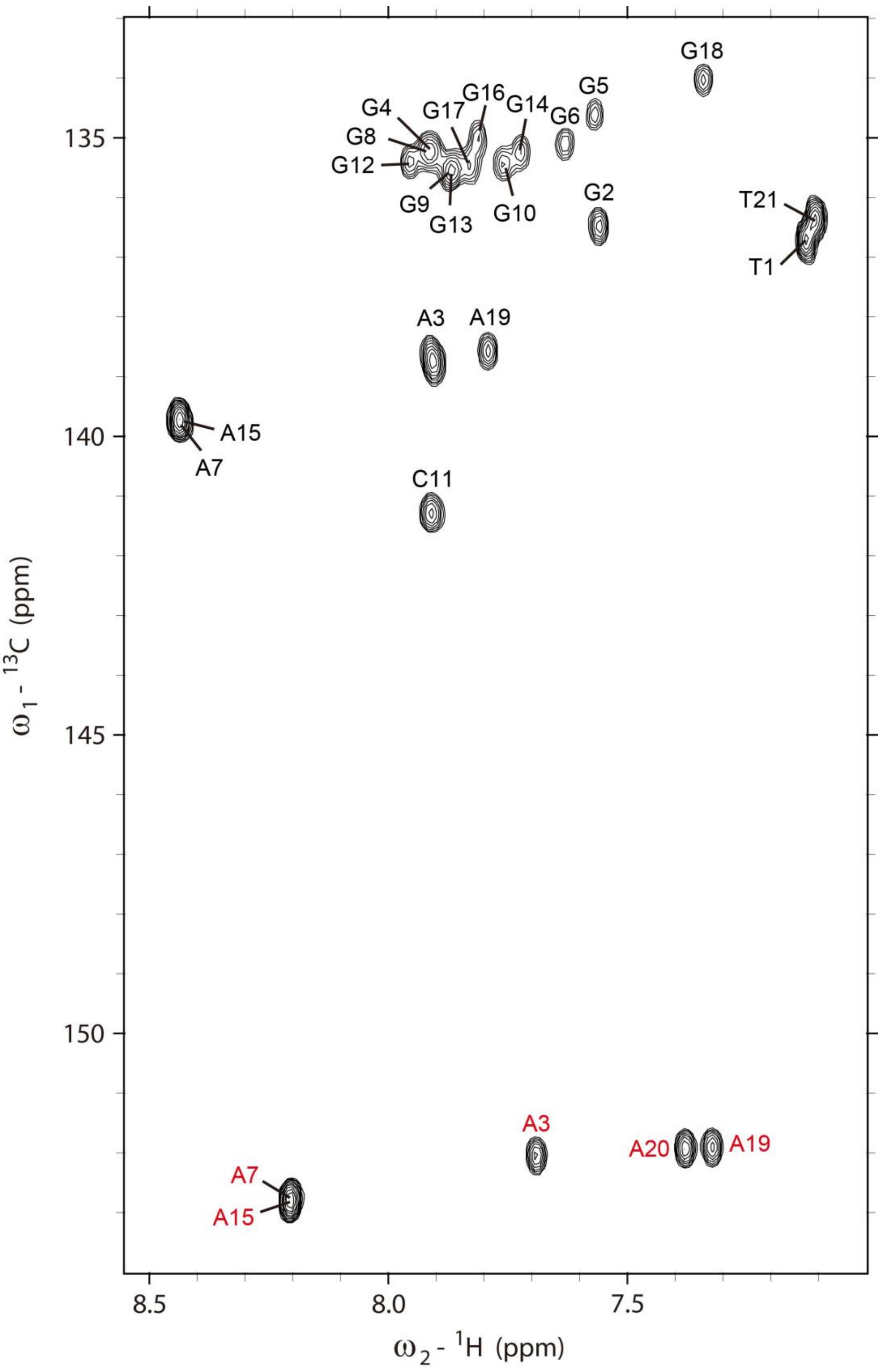

Figure S6. C8−H8/C6−H6 cross-peak assignments for A, G, T, and C bases (black labels), and C2-H2 cross-peak assignments for A bases (red labels) in the HSQC spectrum of free *BLM*-G4. Conditions: 1.93 mM *BLM*-G4, pH 7.0, 50 mM K^+, 25°C.

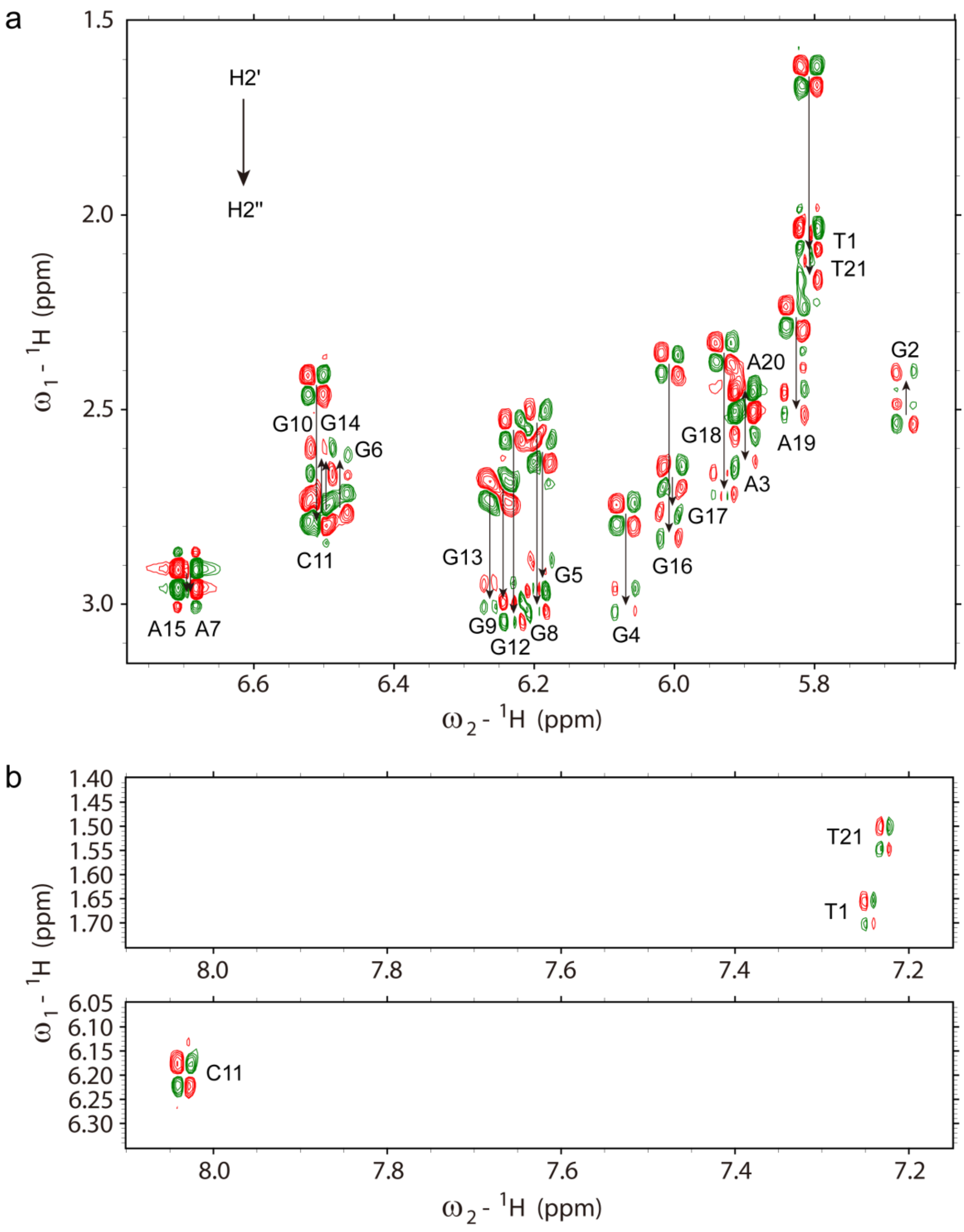

Figure S7. The signal assignments of free *BLM*-G4 in its DQF-COSY spectrum. (**a**) The signal assignments of H2′(H2′′)-H1′ intra-residue cross-peaks in the DQF-COSY spectrum of free *BLM*-G4. The H2′/H2′′ assignments are indicated by the arrow directions. (**b**) The signal assignments of thymine methyl-H6 (top) and cytosine H5-H6 (bottom) cross-peaks in the DQF-COSY spectrum of free *BLM*-G4. Conditions: 1.93 mM *BLM*-G4, pH 7.0, 50 mM K^+, 25°C.

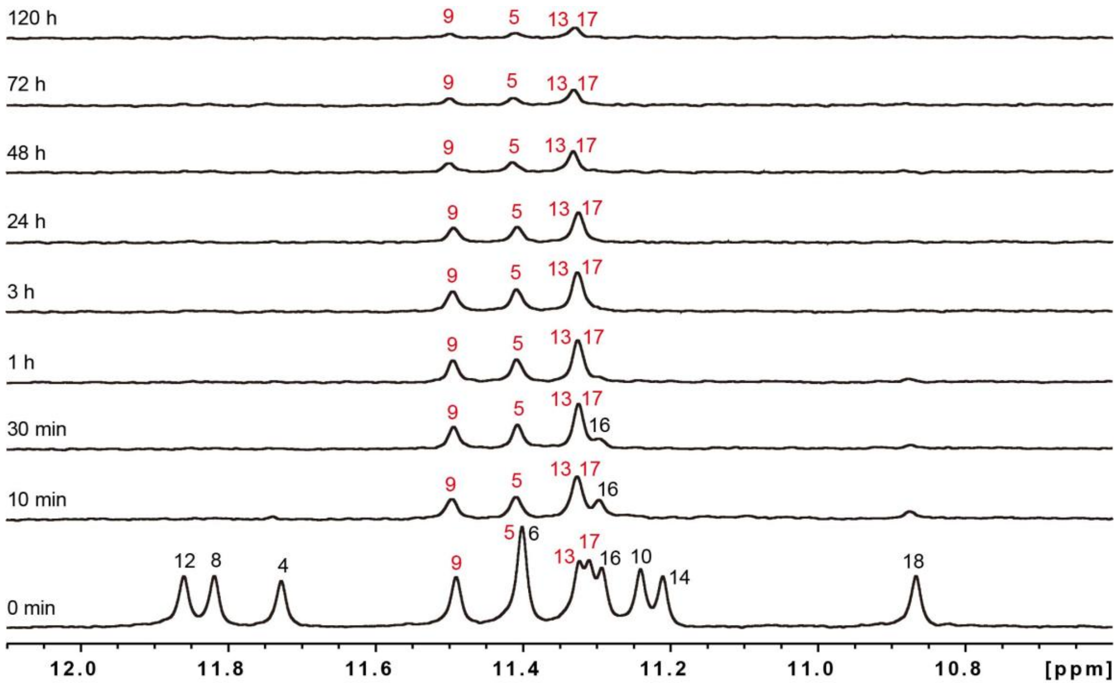

Figure S8. Imino proton spectra of free *BLM*-G4 during different time courses of the D_2O exchange experiment. Conditions: 150 µM *BLM*-G4, pH 7.0, 50 mM K^+ solution, 25°C.

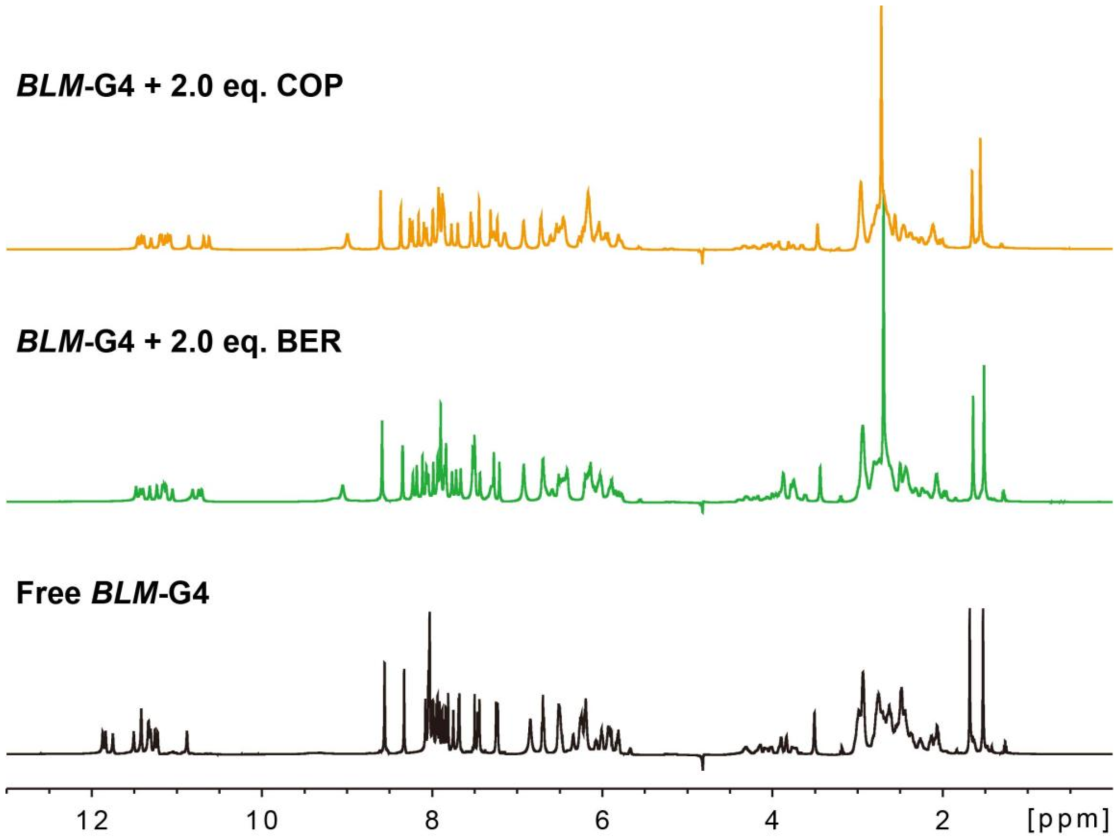

Figure S9. The full ^{1}H NMR spectra of *BLM*-G4 with and without coptisine (COP) or berberine (BER), respectively. Conditions: 1.93 mM *BLM*-G4, pH 7.0, 50 mM K^+ solution, 25°C.

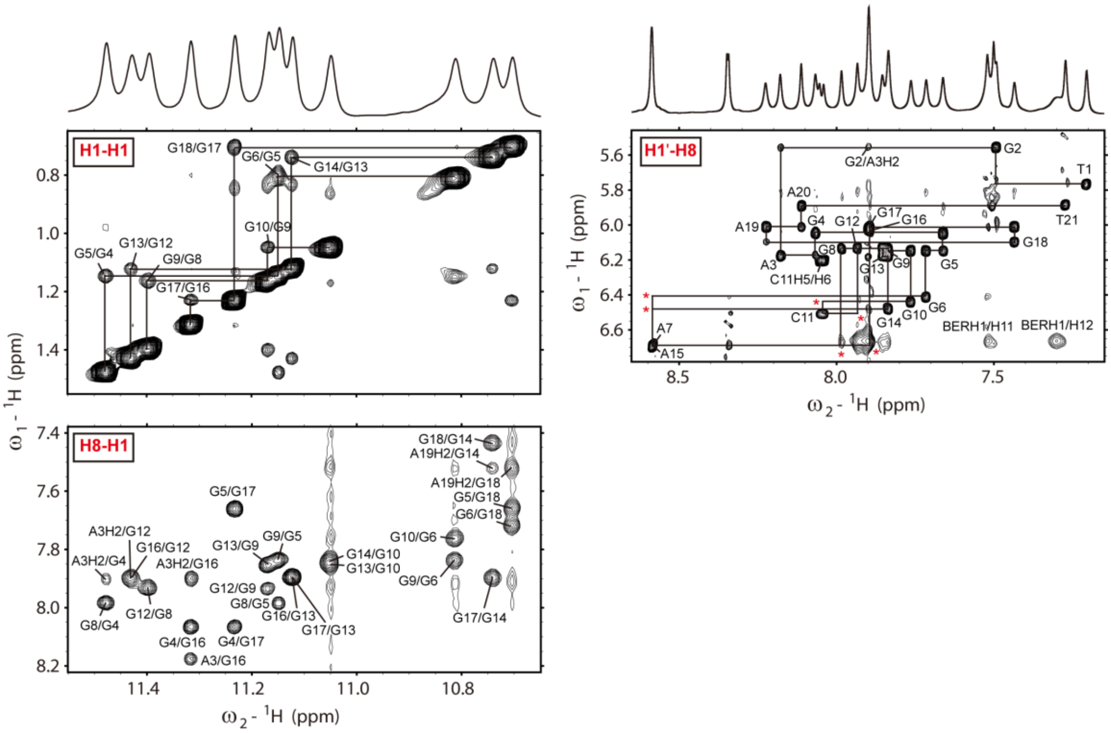

Figure S10. The signal assignments of H1-H1, H8−H1, and H1′-H8 regions from the NOESY spectrum of BER-*BLM*-G4. Conditions: 1.93 mM *BLM*-G4, BER : *BLM*-G4 = 2:1, pH 7.0, 50 mM K^+, 25°C.

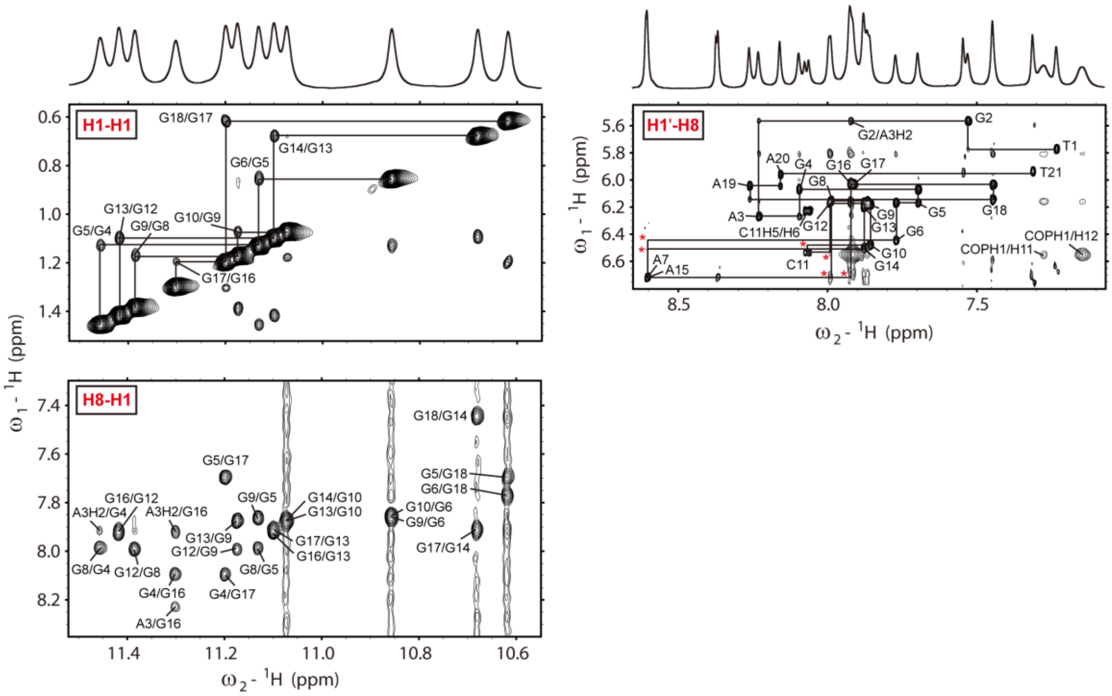

Figure S11. The signal assignments of H1-H1, H8−H1, and H1′-H8 regions from the NOESY spectrum of COP-*BLM*-G4. Conditions: 1.62 mM *BLM*-G4, COP : *BLM*-G4 = 2:1, pH 7.0, 50 mM K^+, 25°C.

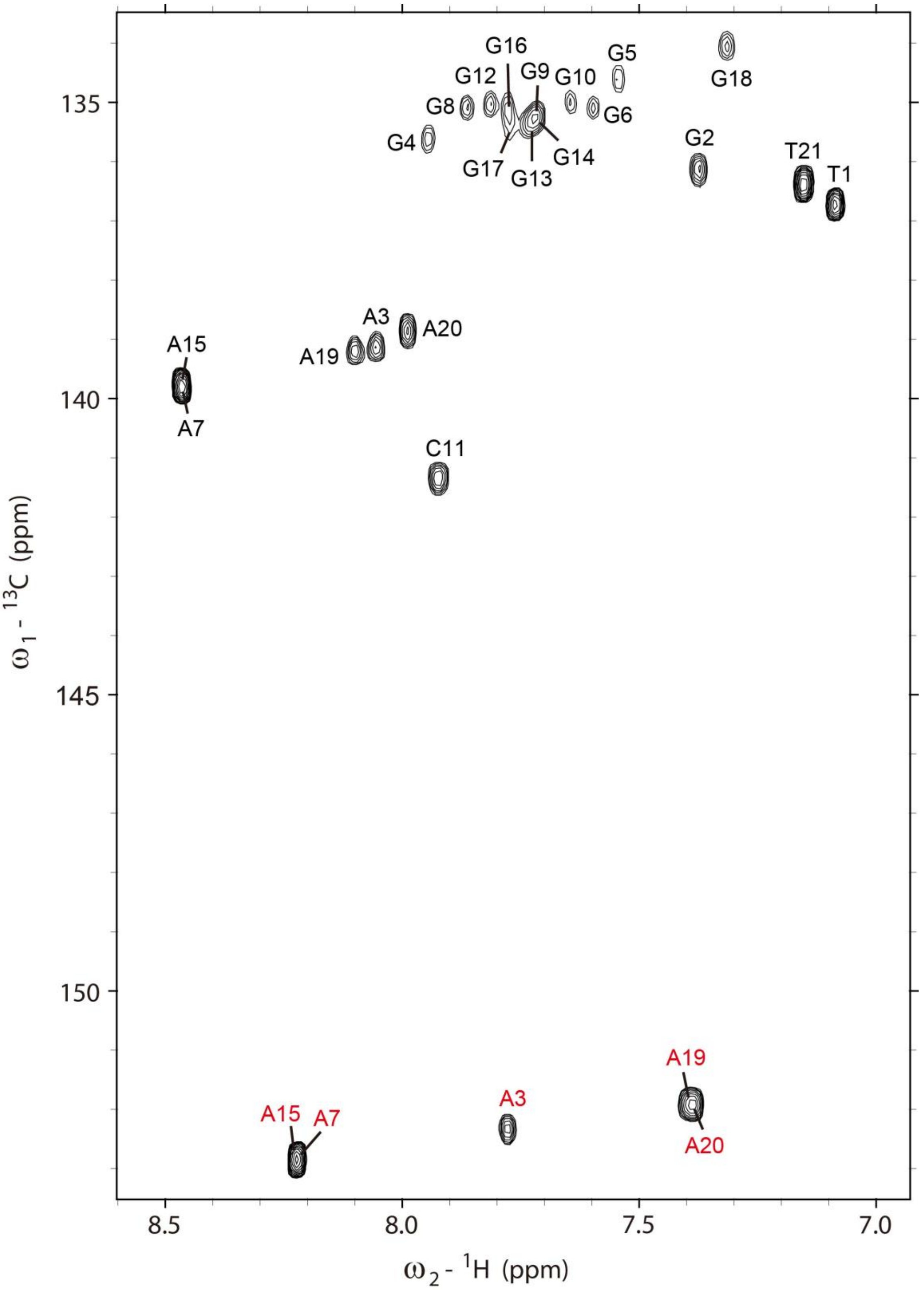

Figure S12. C8−H8/C6−H6 cross-peak assignments for A, G, T, and C bases (black labels), and C2-H2 cross-peak assignments for A bases (red labels) in the HSQC spectrum of BER-*BLM*-G4. Conditions: 1.93 mM *BLM*-G4, BER : *BLM*-G4 = 2:1, pH 7.0, 50 mM K^+, 25°C.

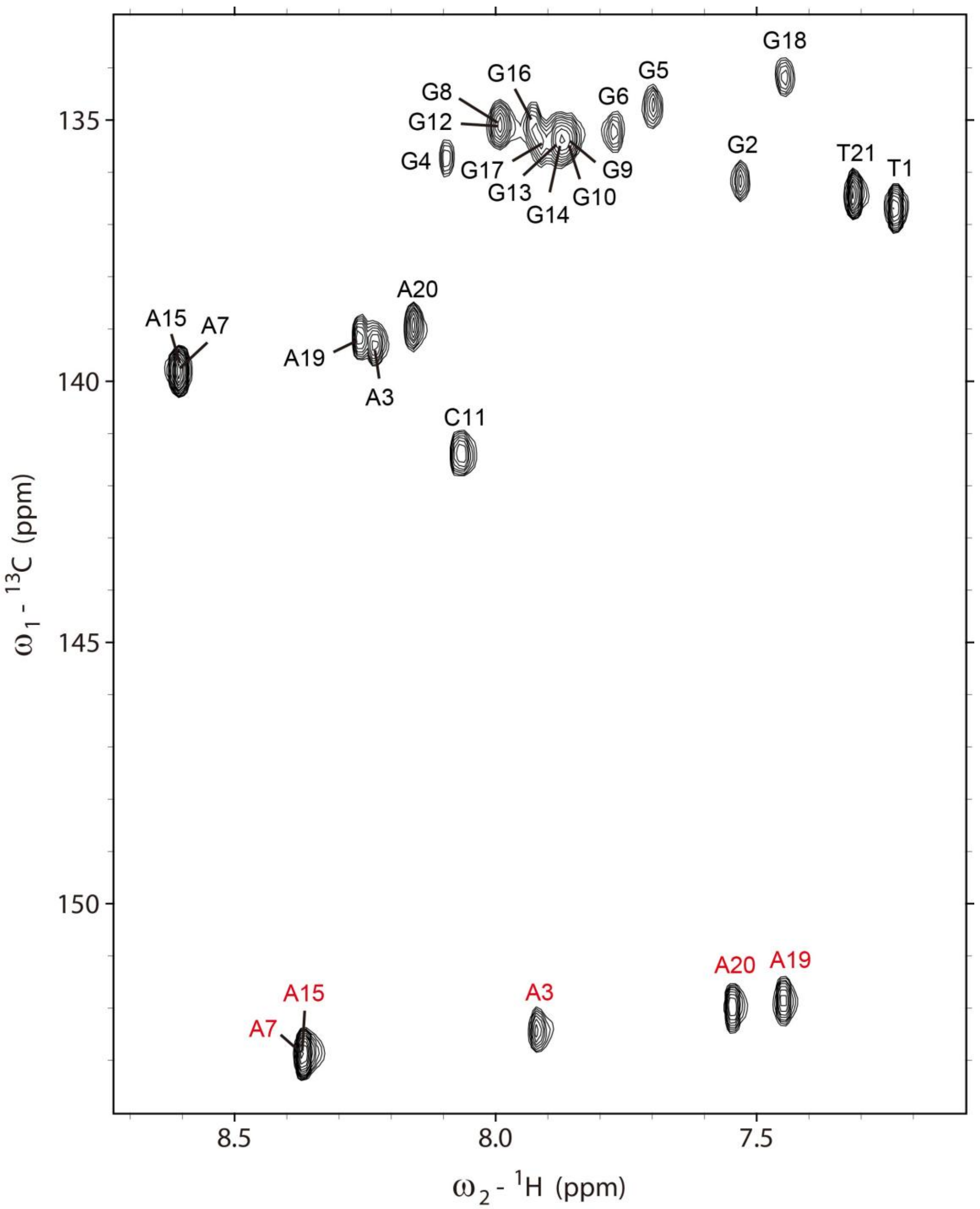

Figure S13. C8−H8/C6−H6 cross-peak assignments for A, G, T, and C bases (black labels), and C2-H2 cross-peak assignments for A bases (red labels) in the HSQC spectrum of COP-*BLM*-G4. Conditions: 1.62 mM *BLM*-G4, COP : *BLM*-G4 = 2:1, pH 7.0, 50 mM K^+, 25°C.

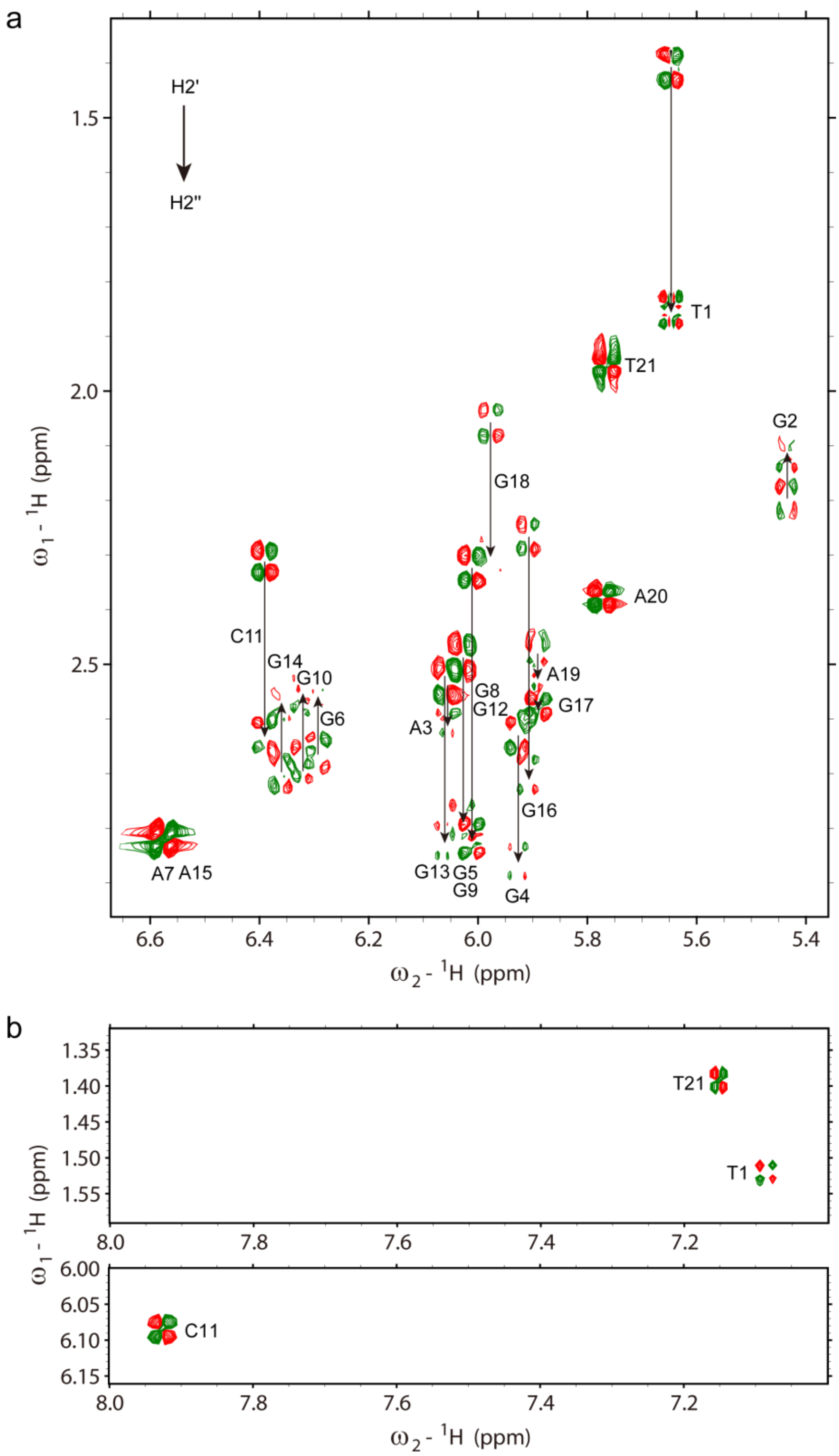

Figure S14. The signal assignments of BER-*BLM*-G4 in its DQF-COSY spectrum. (**a**) The signal assignments of H2′(H2′′)-H1′ intra-residue cross-peaks in the DQF-COSY spectrum of BER-*BLM*-G4. The H2′/H2′′ assignments are indicated by the arrow directions. (**b**) The signal assignments of thymine methyl-H6 (top) and cytosine H5-H6 (bottom) cross-peaks in the DQF-COSY spectrum of BER-*BLM*-G4. Conditions: 1.93 mM *BLM*-G4, BER : *BLM*-G4 = 2:1, pH 7.0, 50 mM K^+, 25°C.

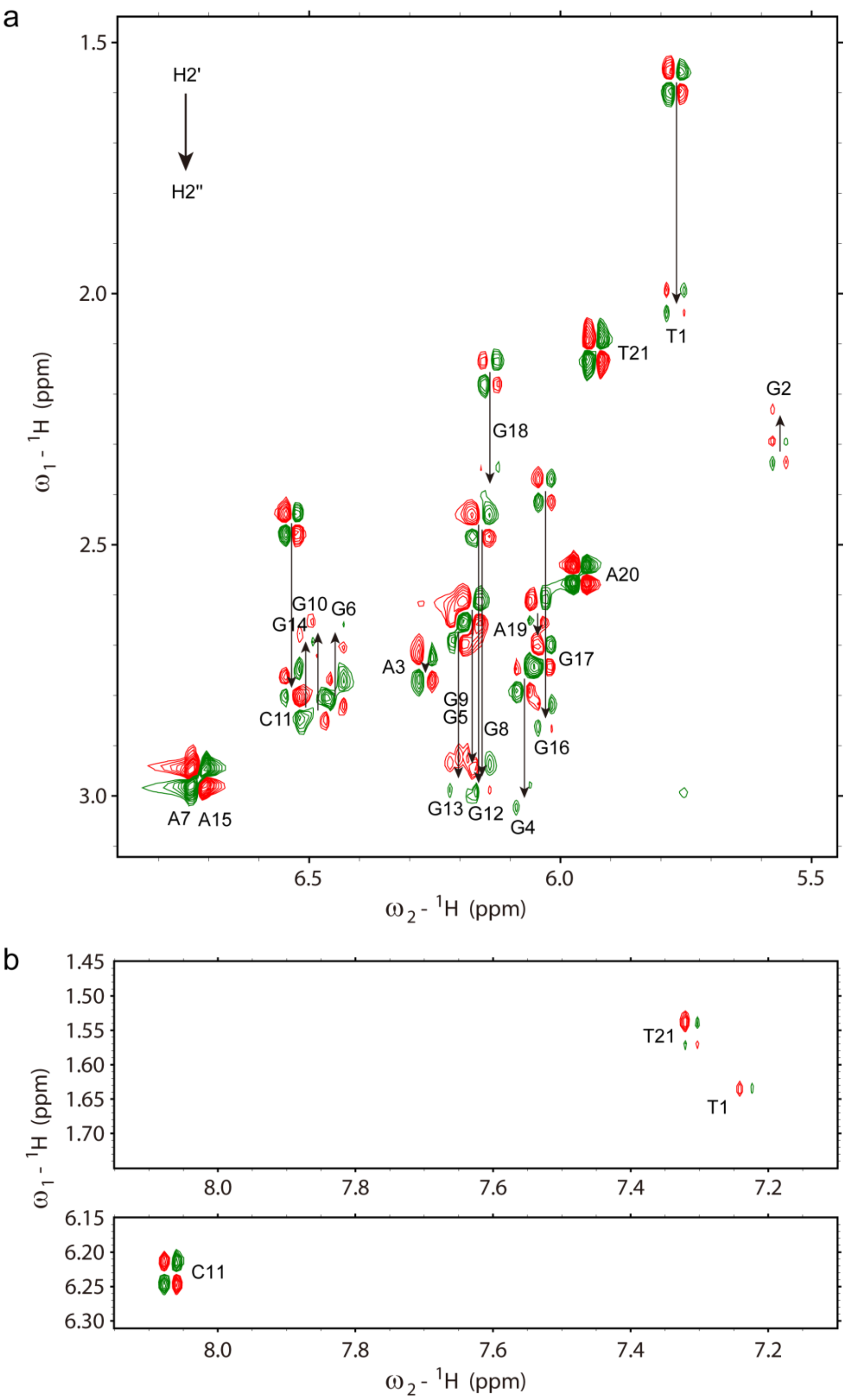

Figure S15. The signal assignments of COP-*BLM*-G4 in its DQF-COSY spectrum. (**a**) The signal assignments of H2′(H2″)-H1′ intra-residue cross-peaks in the DQF-COSY spectrum of COP-*BLM*-G4. The H2′/H2″ assignments are indicated by the arrow directions. (**b**) The signal assignments of thymine methyl-H6 (top) and cytosine H5-H6 (bottom) cross-peaks in the DQF-COSY spectrum of COP-*BLM*-G4. Conditions: 1.62 mM *BLM*-G4, COP : *BLM*-G4 = 2:1, pH 7.0, 50 mM K^+, 25°C.

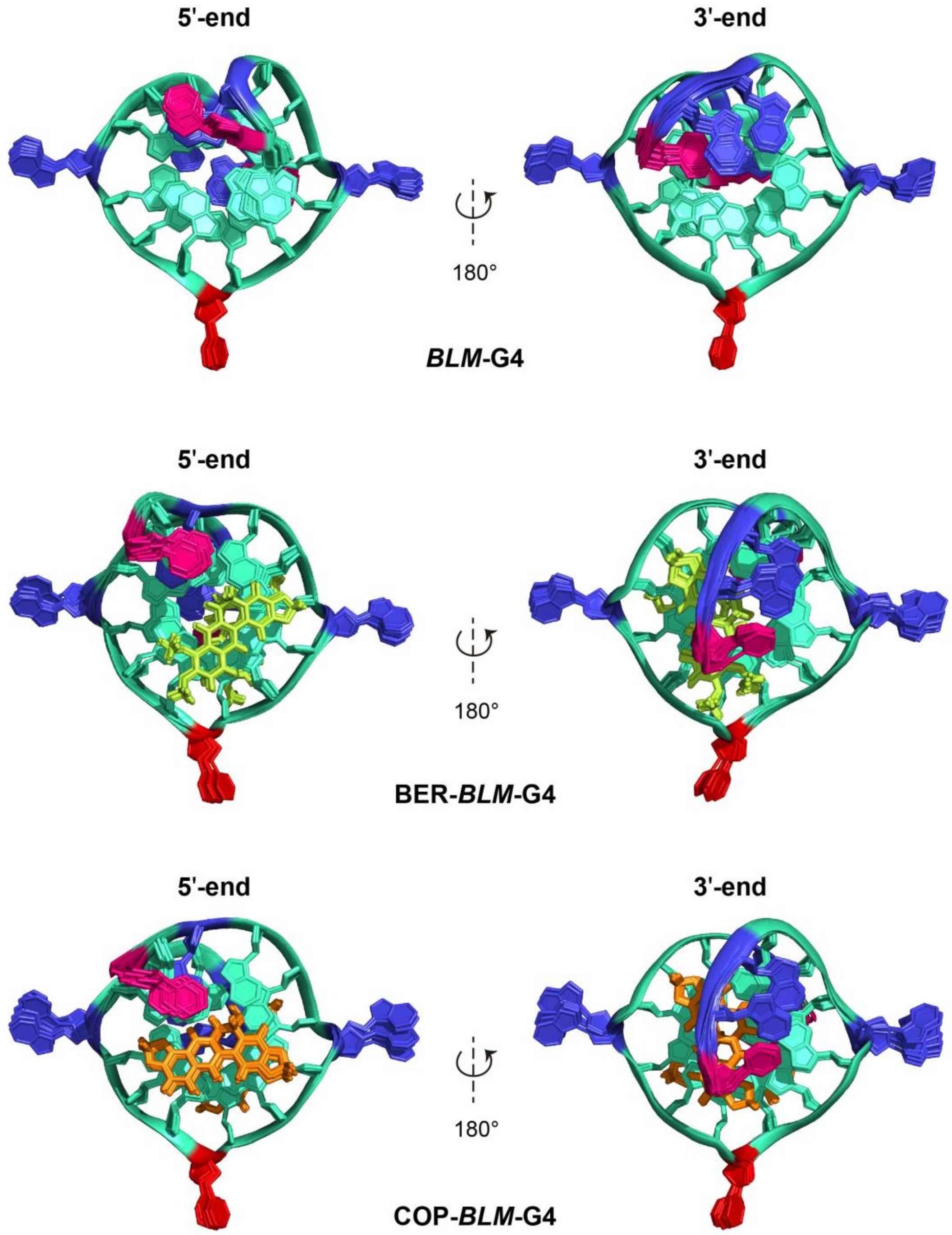

Figure S16. The top and bottom views of 10 overlapped lowest-energy solution structures of *BLM*-G4, BER-*BLM*-G4, and COP-*BLM*-G4, respectively.

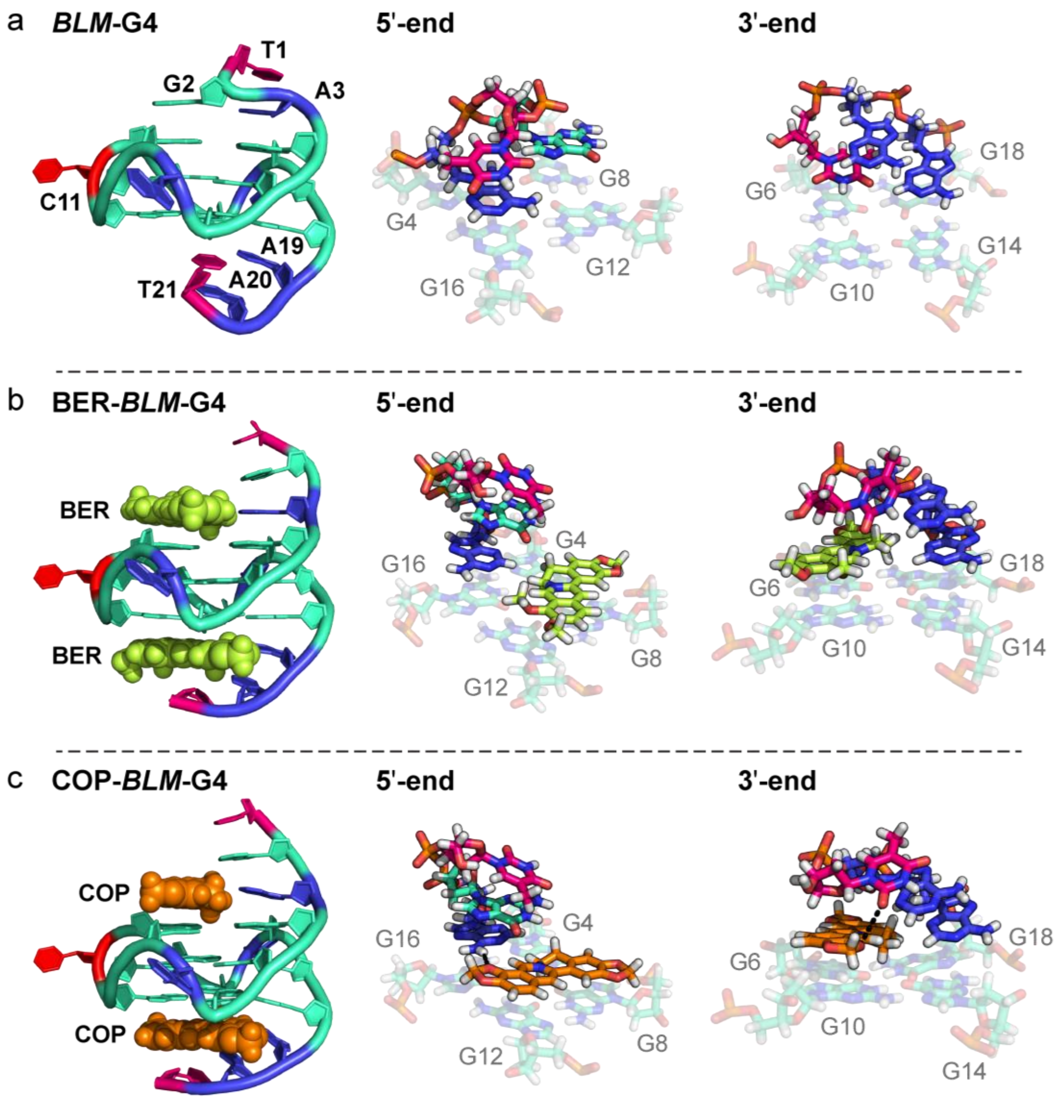

Figure S17. The lateral structures, 5′-end and 3′-end capping structures of *BLM*-G4 (**a**), BER-*BLM*-G4 (**b**), and COP-*BLM*-G4 (**c**).

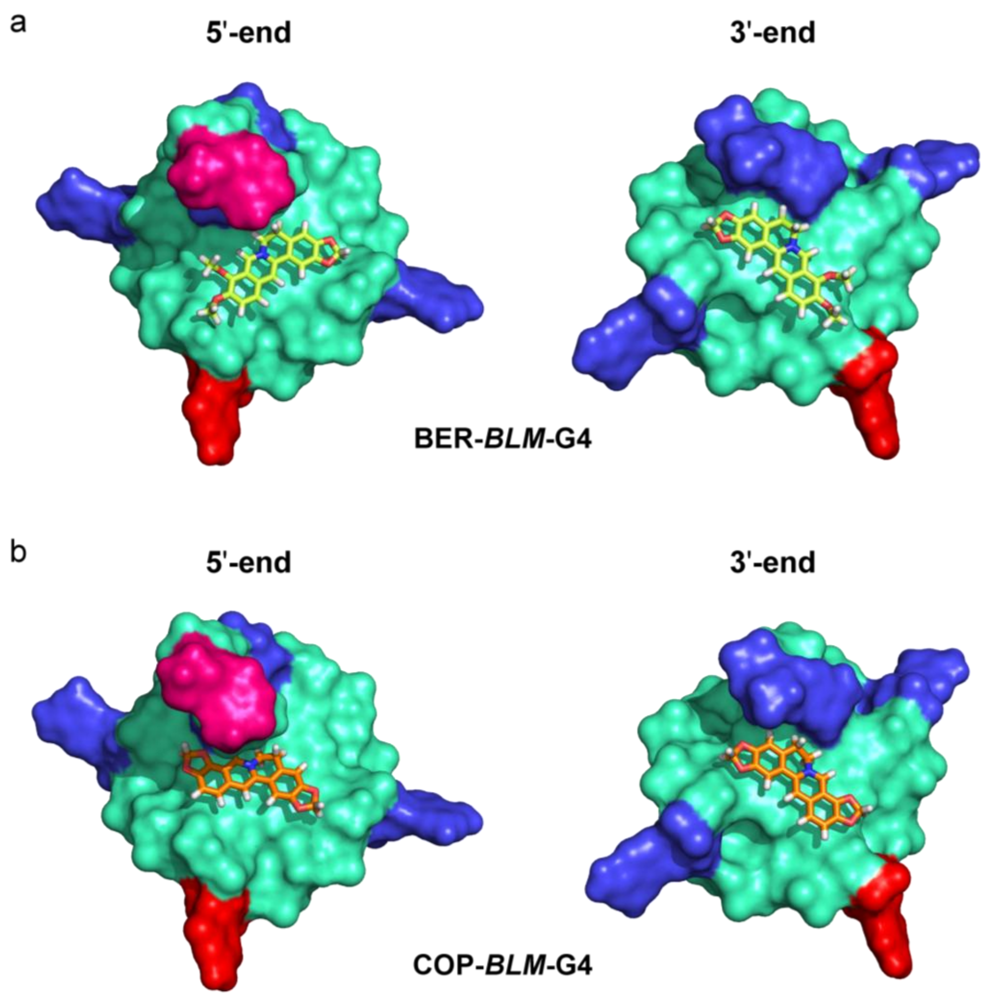

Figure S18. The top and bottom views of BER-*BLM*-G4 (**a**) and COP-*BLM*-G4 (**b**) in surface mode underlying the possible binding grooves of *BLM*-G4 for drug derivatization.

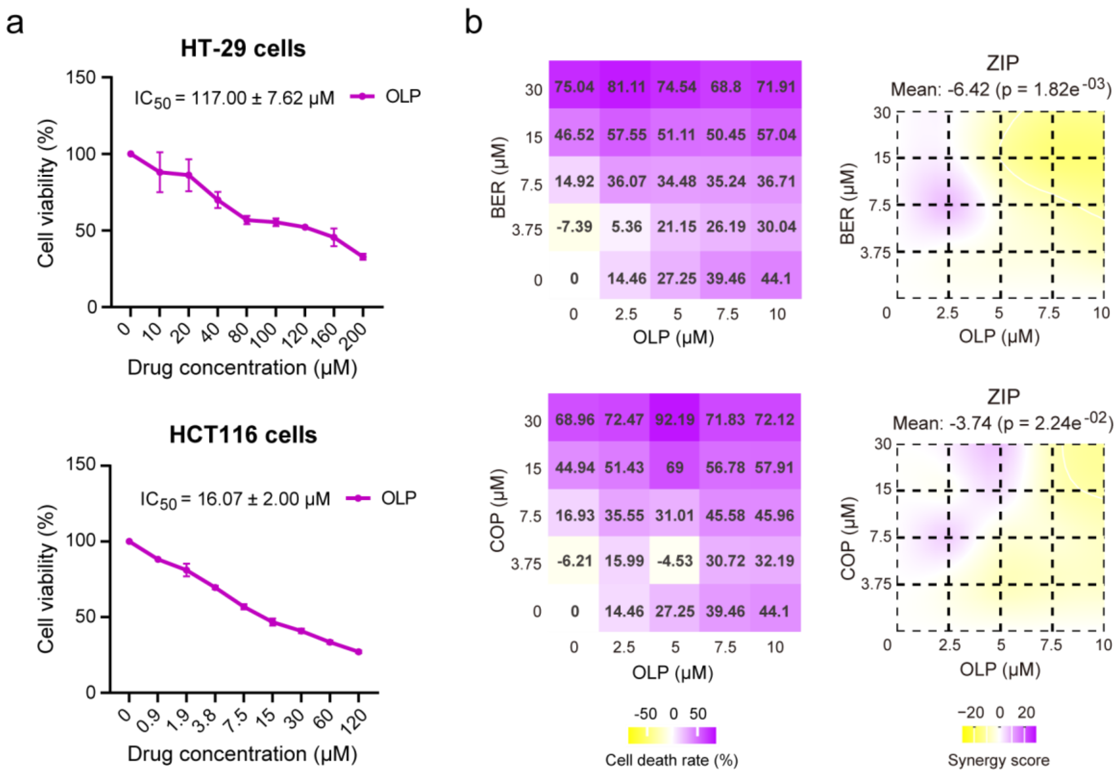

Figure S19. The effects of olaparib (OLP) and drug combination with BER/COP on HT-29 and HCT116 cell viabilities. (**a**) Cell viabilities of HT-29 cells (top) and HCT116 cells (bottom) under the treatment with OLP for 72 h, as determined by CCK-8. Statistical data were shown as means ± standard deviation of at least three independent experiments. (**b**) The cell death rates (left) and the corresponding ZIP synergy plots (right) of HCT116 cells under the treatment with different drug combinations for 72 h. Cell death rates were shown as means of three independent experiments.

References

1. Wang, K.B., Liu, Y., Li, J., Xiao, C., Wang, Y., Gu, W., Li, Y., Xia, Y.Z., Yan, T., Yang, M.H., *et al.* (2022) Structural insight into the bulge-containing KRAS oncogene promoter G-quadruplex bound to berberine and coptisine. *Nat. Commun.*, **13**, 6016.
2. Liu, Y., Li, J., Zhang, Y., Wang, Y., Chen, J., Bian, Y., Xia, Y., Yang, M.H., Zheng, K., Wang, K.B., *et al.* (2023) Structure of the Major G-Quadruplex in the Human EGFR Oncogene Promoter Adopts a Unique Folding Topology with a Distinctive Snap-Back Loop. *J. Am. Chem. Soc.*, **145**, 16228-16237.
3. Huang, Z., Liu, H., Nix, J., Xu, R., Knoverek, C.R., Bowman, G.R., Amarasinghe, G.K. and Sibley, L.D. (2022) The intrinsically disordered protein TgIST from Toxoplasma gondii inhibits STAT1 signaling by blocking cofactor recruitment. *Nat. Commun.*, **13**, 4047.
4. Vinkemeier, U., Cohen, S.L., Moarefi, I., Chait, B.T., Kuriyan, J. and Darnell, J.E., Jr. (1996) DNA binding of in vitro activated Stat1 alpha, Stat1 beta and truncated Stat1: interaction between NH2-terminal domains stabilizes binding of two dimers to tandem DNA sites. *EMBO J.*, **15**, 5616-5626.
